# Assessing the invasive potential of different source populations of ragweed (*Ambrosia artemisiifolia* L.) through genomically informed species distribution modelling

**DOI:** 10.1111/eva.13632

**Published:** 2023-12-20

**Authors:** Andhika R. Putra, Kathryn A. Hodgins, Alexandre Fournier‐Level

**Affiliations:** ^1^ School of BioSciences The University of Melbourne Parkville Victoria Australia; ^2^ School of Biological Sciences Monash University Clayton Victoria Australia

**Keywords:** biosecurity, ecological niche model, genomics, invasive species, weed

## Abstract

The genetic composition of founding populations is likely to play a key role in determining invasion success. Individual genotypes may differ in habitat preference and environmental tolerance, so their ability to colonize novel environments can be highly variable. Despite the importance of genetic variation on invasion success, its influence on the potential distribution of invaders is rarely investigated. Here, we integrate population genomics and ecological niche models (ENMs) into a single framework to predict the distribution of globally invasive common ragweed (*Ambrosia artemisiifolia*) in Australia. We identified three genetic clusters for ragweed and used these to construct cluster‐specific ENMs and characterize within‐species niche differentiation. The potential range of ragweed in Australia depended on the genetic composition and continent of origin of the introduced population. Invaders originating from warmer, wetter climates had a broader potential distribution than those from cooler, drier ones. By quantifying this change, we identified source populations most likely to expand the ragweed distribution. As prevention remains the most effective method of invasive species management, our work provides a valuable way of ranking the threat posed by different populations to better inform management decisions.

## 1 INTRODUCTION

Introductions of alien species show no signs of abating globally. Introduction rates have continued to increase since the 19th century, declining only briefly following World War II (Bonnamour et al., 2021; Hulme, 2009; Seebens et al., 2017; van Kleunen et al., 2015). Although the Tens Rule (Williamson & Fitter, 1996) suggests only 0.1% of introductions produce invasive species capable of spreading without human assistance (Richardson et al., 2000), this small fraction can significantly threaten biodiversity, ecosystem functioning, and human health (Gurevitch & Padilla, 2004; Kumar Rai & Singh, 2020; Oswalt & Marshall, 2008; Tobin, 2018).

Preventing introductions remains the most effective method of invasive species management (Keller et al., 2008; Leung et al., 2002). Prophylactic strategies include surveillance of invasive species, identifying potential introduction routes, and understanding the factors shaping species' biogeography (Blossey, 1999; Hulme, 2009; Lecocq et al., 2016). In the case of invasive plants, multiple environmental, biological and anthropogenic factors can influence their distribution. These include the existence of trade routes, the mating system of invaders, escape from native pathogens and predatory pressure in the introduced range, and their adaptive potential in novel environments (Chapman et al., 2016; Flory & Clay, 2013; Grant & Kalisz, 2020; Hulme, 2009; Peterson et al., 2008; Scalone et al., 2016; Sheth et al., 2020).

Predicting the potential range of an invader is a risk assessment strategy that allows managers to allocate resources according to the susceptibility of different sites to invasion. However, the early stages of invasion will see invaders colonizing only a fraction of all suitable habitat. This biases the realized niche towards environments found near the introduction zone. If this niche bias is confounded with habitat preference, the species' potential distribution may be underestimated (Václavík & Meentemeyer, 2012).

Overcoming this bias requires considering regions where species distributions are in equilibrium with their environment, being present in all suitable environments and absent from unsuitable ones. Equilibrium is often assumed in the native range (Guisan & Thuiller, 2005), but can also occur in older introduced ranges. In these older introduced ranges, invasion may have been followed by niche shifts that expand the species‐wide realized niche (Atwater et al., 2018; Broennimann et al., 2007).

Occurrence records from regions where species are at equilibrium can be used to build ecological niche models (ENMs; Peterson, 2006) that predict the potential distribution of invasive species (Barbet‐Massin et al., 2018; Briscoe Runquist et al., 2019; Rasmussen et al., 2017; Sung et al., 2018). However, there is no universal solution to the challenge of identifying regions at risk of invasion. In fact, it would be misleading to suggest that the use of global distribution data is inherently more informative – some models calibrated on native‐range occurrence data can have questionable transferability to the introduced ranges (Liu et al., 2020) and information on intraspecific niche differentiation may be lost if all records are considered simultaneously in a single model. The latter is particularly important to consider because invasions often begin with a few founding individuals. The genetic composition of these founders can significantly impact invasion success by either constraining species distributions (Blackburn et al., 2015; Wellband et al., 2018; Zenni & Nuñez, 2013) or facilitating adaptive evolution and range expansion (Chapman et al., 2017; Hällfors et al., 2016; Moran & Alexander, 2014; Rodríguez‐Rodríguez et al., 2020).

Factoring the genetic make‐up of founder individuals during invasion parallels the importance of appropriate record selection during ENM construction (Guillera‐Arroita et al., 2015), as both influence the potential species distributions. An important question to address is how to characterize intraspecific niche differentiation when modelling species distribution. This can be accomplished by the inclusion of genetic information into ENMs. The relationship between genetic and environmental variation is well‐established, with studies highlighting the effect of environmental selection on genetic variation across various taxa (Bhattarai et al., 2017; Fournier‐Level et al., 2011). Patterns of genetic diversity, in turn, strongly influence species distribution because genetic differences determine both adaptive potential and environmental tolerance in the introduced range (Willi et al., 2006). However, questions remain as to the effect of different genetic make‐ups on an invader's potential distribution. How does varying source population affect the potential range of a species? What role do different sources of genetic variation have on the invasive potential of an existing population of invaders? The former has been addressed in endemic but not in invasive species (Hu et al., 2021; Razgour et al., 2019); the latter is usually done with a focus on establishment success rather than potential distribution (Ahlroth et al., 2003; Blackburn et al., 2015; Lockwood et al., 2009).

A key feature for investigating the role of genetic variation on invasive species distributions is the occurrence of multiple invasion events across distinct geographic areas. In this regard, common ragweed (*Ambrosia artemisiifolia*) is a powerful model system. This annual, strictly outcrossing plant has a well‐characterized genome (2*n* = 36) of approximately 1.1 Gbp (Battlay et al., 2023). Ragweed is native to North America but has become globally invasive, occurring in parts of Asia, Africa, Australia, South America, and Europe (Essl et al., 2015). Wherever present, ragweed pollen is a costly public health hazard (Schaffner et al., 2020) while ragweed plants are sometimes agricultural weeds in soy and white bean cropping (Chikoye et al., 1995; Coble et al., 1981). Its success can be attributed to the seeds' ability to contaminate goods, co‐opt human cargo as transport vessels (Dahl et al., 1999; Laaidi et al., 2003; Lemke et al., 2021), and rapidly colonize disturbed habitats (Gentili et al., 2017). In Australia – our introduced range of interest – ragweed has been observed since at least the 1930s (Bass et al., 2000) and is naturalized in coastal Queensland and New South Wales (GBIF Secretariat, 2023).

Across its native North American and older invasive European ranges, ragweed is assumed to be at equilibrium owing to its ubiquity. In contrast, Australian ragweed is restricted to the eastern parts of the continent. Ragweed is absent from the more tropical northern Australia despite being naturalized in places like Colombia and Hawaii (van Kleunen et al., 2015). Further south, occurrences conspicuously stop along the Victoria – New South Wales state border. This restricted distribution may be a result of common ragweed co‐occurring with the similar‐looking *Parthenium hysterophorus* (‘false ragweed’), a Weed of National Significance (Atlas of Living Australia, 2023) that is carefully managed. The management of ragweed in Australia contrasts with historical examples of ragweed expansion that was facilitated elsewhere by the absence of biosecurity measures (Martin et al., 2014; Oswalt & Marshall, 2008) and thus suggests weed management was successful in limiting its distribution in Australia. Taken together, this suggests the Australian ragweed population is not at equilibrium. Thus, knowing its potential distribution will be a valuable and cost‐effective asset for ragweed management (Katz et al., 2014; Richter et al., 2013).

In this study, we use genomic variation to improve the prediction of ragweed's potential distribution in Australia. Specifically, we aimed to leverage genomic data obtained from a global sampling to determine the effect of varying genetic composition on ragweed potential distribution. We focused on addressing three main aims. Firstly, we tested if ragweed showed niche differentiation among continents. If Australian ragweed presently occupies a narrower set of environmental conditions than elsewhere, this would suggest the species' potential distribution on the continent is broader than currently observed. Secondly, we investigated the relationship between ragweed genomic variation and habitat preferences. Previous work found local adaptation to be prevalent in ragweed, with a statistically significant correlation between genetic and environmental variation for putatively adaptive loci (Battlay et al., 2023; van Boheemen et al., 2019; van Boheemen & Hodgins, 2020). However, whether this relationship is seen across the whole genome and how this impacts the species distribution remains unclear. Finally, we assessed the effect of varying genetic composition of founders on distribution in the novel introduced range of Australia. The consequence of different founder compositions on invasion success is rarely investigated (Zenni & Nuñez, 2013), despite local adaptation suggesting genetic composition will impact distribution. We compared scenarios based on different invasion sources to determine its effect on the potential distribution of ragweed and assess the relative threat of range expansion in Australia posed by introduction of plant propagules from different source ranges.

## 2 MATERIALS AND METHODS

### 2.1 Global occurrence records

Occurrence records for *A. artemisiifolia* were obtained from the Global Biodiversity Information Facility (GBIF.org, 2023). Records were filtered to retain only ‘observations’ or ‘human observations’ with coordinate uncertainty less than 50 km and duplicates with identical coordinates were removed. Records were then thinned to one observation per 2.5′ × 2.5′ cell (approximately 4.5 x 4.5 km at the equator) using the gridSample function in R\raster (Hijmans et al., 2020), resulting in 27,472 records globally. Global records were further distinguished into records from the native North American (11,694 records), older introduced European (15,086 records) and more recent introduced Australian (440 records) ranges.

### 2.2 Genomic data

We used a combination of whole‐genome sequencing (WGS) and Genotyping‐by‐Sequencing (GBS) to obtain data for ragweed genomic variation. WGS data was obtained for 311 individuals sampled between 2007 and 2019 across ragweed's modern North American and European range (Bieker et al., 2022) leading to the genotyping of 8,023,263 Single Nucleotide Polymorphisms (SNPs) across 30 scaffolds. GBS data was obtained for 860 *A. artemisiifolia* plants sourced during the 2013–2014 growing season from North America, Europe, and Australia (van Boheemen et al., 2019).

Sequencing reads obtained through WGS and GBS were aligned to the *A. artemisiifolia* reference genome (Battlay et al., 2023) using bwa mem (Li, 2013) and then ran through the PALEOMIX pipeline (Schubert et al., 2014). PCR duplicates among the WGS sequencing reads were identified using the bammarkduplicates function from the biobambam package (Tischler & Leonard, 2014) and removed using picard (http://broadinstitute.github.io/picard/). Single Nucleotide Polymorphisms (SNPs) were called using HaplotypeCaller from the GATK4 package (Poplin et al., 2018). The SNP data from the 311 individuals genotyped through WGS was used as reference for the imputation of missing genotypes in the GBS dataset using BEAGLE 5.2 (Browning et al., 2018). Scaffolds without markers overlapping between the WGS and the GBS set were excluded, retaining 5,396,964 imputed SNPs across 20 scaffolds. The imputed SNPs were filtered to only select those with no missing calls and any SNPs with a minor allele frequency lower than 0.05 removed. This resulted in a final dataset of 27,084 SNPs genotyped across 860 individuals. All genotyped individuals occurred within the reported distribution of ragweed in GBIF (Figure S1).

### 2.3 Model design

ENMs that did not consider the genetic composition of founders were designed in the R statistical software v3.6.3 by fitting maximum entropy models (MaxEnt; Phillips et al., 2006) as implemented in the R/predicts package (Hijmans, 2023) using records from GBIF.org. MaxEnt was chosen because of its consistent accuracy and broad use (Table S1; Merow et al., 2013; Valavi et al., 2022), making it the most straightforward framework for demonstrating the value of incorporating genomic data into ENMs. We created separate models for each of the three ranges (North America, Europe, Australia) using bioclimatic variables sourced from WorldClim v2 (Fick & Hijmans, 2017) and land use/cover variables sourced from the Harmonized World Soil Database (v1.2; Fischer et al., 2008) as predictors (Table S2). Bioclimatic variables were chosen as they describe climatic conditions of sites, while land use/cover was included because ragweed strongly prefers highly disturbed and open habitats (Essl et al., 2015). The resolution of predictors was homogenized by resampling land use/cover to a 2.5′ x 2.5′ resolution using the resample() function from the R\raster package (Hijmans et al., 2020).

Model goodness‐of‐fit was assessed using AUC (area under the Receiver‐Operator curve), a threshold‐independent metric commonly used to measure the performance of MaxEnt models (Merow et al., 2013). MaxEnt includes a regularization step to prevent model overfitting. We tested four levels of regularization (betamultiplier = {0.01,1,5,10}) and validated the use of 1 as regularization multiplier based on AUC of models for Australia. For each model, we created a 1′ buffer around occurrence records and sampled 10,000 randomly selected range‐specific background points outside the buffer region to define the available niche. The breadth of the realized niche was visualized using principal component analysis (PCA). Predictions of potential distribution were created by projecting the fitted model to the entire continent of Australia.

### 2.4 Genetic clustering

We incorporated genomic data into ENMs by assigning plants to different genetic clusters according to ancestries estimated using ADMIXTURE (v1.3.0; Alexander & Lange, 2011). The number of ancestral populations was determined by running ADMIXTURE on genomic data from native North American plants. We selected *L = 3* ancestral populations based on both the minimization of the 5‐fold cross‐validation error and the rate of change in model likelihood (Table S3, Figure S2, Evanno et al., 2005). Ancestries were then estimated for the European and Australian individuals using ADMIXTURE's projection function. Finally, the inferred ancestries were used to separate plants into *k = 3* clusters via *k*‐means clustering. A low value for *k* ensured clusters were present across all ranges to enable within‐range estimation of niche overlap among clusters, as higher values would cause certain range‐cluster combinations to be absent. Finally, we characterized the pattern of genetic differentiation between clusters by estimating the fixation index *F*_*ST*_ using vcftools 0.1.16 (Danecek et al., 2011).

### 2.5 Assessing habitat preference for the different clusters

Genetic and environmental variations are often correlated, such that genetically distinct clusters occur in different environments. However, this pattern may be disrupted when individuals are randomly introduced to a new range. To determine whether environmental niches are conserved between genetically similar individuals found in different ranges or if the relationship between genetic and environmental variation is disrupted following introduction events, we estimated niche overlap between model projections using Schoener's *D*. (Warren et al., 2008). For a pair of ENMs *M*_*X*_ and *M*_*Y*_, Schoener's *D* (Schoener, 1968) is defined as:

$$D\left(P_{X},P_{Y}\right)=1-\frac{1}{2}\sum_{i}\left|P_{X,i}-P_{Y,i}\right|,$$

where *P*_*X*_,_i_ and *P*_*Y*_,_*i*_ are the scaled probability of presence *M*_*X*_ and *M*_*Y*,_ respectively, estimated through MaxEnt over the *i* cell*s* of the gridded landscape. *D* is a scaled similarity index that ranges from 0 (no overlap between models) to 1 (full overlap). We estimated *D* for each pairwise range combination using the nicheOverlap() function of the R\dismo package (Hijmans et al., 2021). *D* values are sensitive to the choice of background points used to define the available environment (Merow et al., 2013), such that values of *D* estimated between ranges have the potential to reflect differences in habitat availability than habitat preference. To ensure *D* estimates are true measures of overlapping habitat preference, we implemented the test of niche similarity described in Warren et al. (2008). Given two ranges *A* and *B*, each with *n*_*A*_ and *n*_*B*_ observations, two ENMs can be created: *M*_*A*_ is fitted using the *n*_*A*_ true observations in range *A*, whereas *M*_*Brandom*_ uses *n*_*B*_ randomly selected points in range *B*. The two models are used to predict habitat suitability in the range of interest (in this study, Australia) and their outputs used to estimate *D*. This process is repeated for 100 random sets of *n*_*B*_ observations, then performed for the complementary combination (*M*_*Arandom*_ and *M*_*B*_) to produce a null distribution of *D* values. The observed value of *D* was compared to this null distribution and considered significantly different than expected by chance if sitting in the bottom 2.5%‐ile or top 97.5%‐ile of this null distribution. Significant results indicate the estimated niche overlap cannot be explained by differences in habitat availability alone. We also performed a Mantel test comparing the estimates of Schoener's *D* and F_ST_ values to determine whether genetic similarity and niche overlap were correlated. Finally, we characterized variation in habitat preference between clusters by testing the difference in means for bioclimatic and land use/cover variables for the different range and genetic cluster combinations using pairwise Welch's *t‐*test.

### 2.6 Quantifying the effect of introducing new source of genetic variation

For each range‐by‐cluster combination (mean number of observations = 95.56, range of observations = 2–176; Table S4), we fitted models using occurrence records specific to the combination and used the corresponding range as the source of 10,000 random background points. The fitted models were then used to predict habitat suitability in Australia. Predictions of habitat suitability were used to identify the regions most susceptible to invasion and determine the source populations of greatest concern. We quantified the impact of introducing genetic variation from different regions into Australia by measuring its effect on ragweed range size. This was accomplished by quantifying the percentage of Australia predicted to be unsuitable by models fitted to Australian records that became suitable following the introduction of novel genetic variation. We applied a function that determines the number of converted cells for a given threshold z within the [0:1] range and integrated the resulting curve to derive the area. This method creates a threshold‐independent metric that avoids the challenges of selecting an appropriate threshold (Guillera‐Arroita et al., 2015). The estimated area is interpreted as the average increase in number of suitable grid cells under the model and was divided by the total number of grid cells in the landscape to obtain a percentage. We chose to quantify impact through range expansion rather than absolute area because the colonization of novel habitats is more concerning than the introduction of plants to already established populations.

The projection of ENMs into a novel range will involve predicting habitat suitability in novel conditions. In order to assess the extent to which conditions in Australia fell outside the values observed in the training data, an MOP analysis was conducted (mobility‐oriented parity; Owens et al., 2013). For either the group of bioclimatic predictors considered most important by MaxEnt or land use/cover predictors (Table S2), we generated MOP maps using the R\mop package (Cobos et al., 2023) by defining the reference layer as conditions within a 1′ buffer around ragweed occurrences and the projection layer as the entirety of Australia. We retrieved ‘simple’ maps from the MOP projections to determine the number of predictors with values that fell outside the range of conditions observed during model fitting.

## 3 RESULTS

### 3.1 Performance of models blocked by range

**FIGURE 1:**
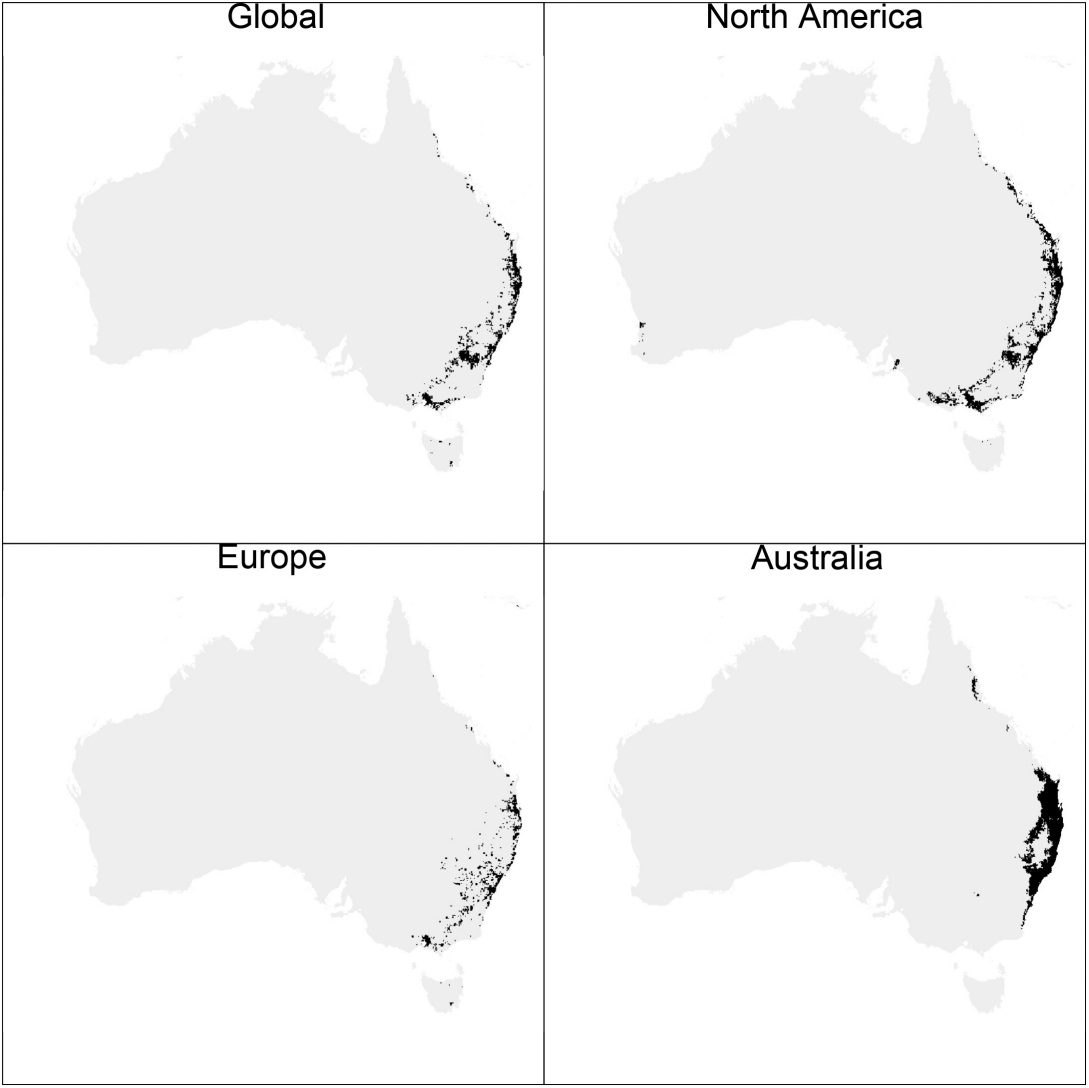
Predicted distribution of *Ambrosia artemisiifolia* in Australia based on observations from the global, North American, European, or Australian range. For each range, a MaxEnt model was created using 19 bioclimatic and 8 land use/cover variables, 10,000 background points, and presence records from GBIF. For ease of visualization, the continuous MaxEnt output was binarized using a threshold value that maximizes both the true positive and true negative rate. The model using global records (All) also includes observations from outside the three ranges considered.

The current distribution of ragweed in Australia was modelled accurately (Figure 1) with consistently high AUC ranging from 0.866 for the model using European observations for training to 0.997 for models using Australian observations (Table S5). However, the predicted distribution differed according to the set of observations used during model training. The model trained on Australian records predicted suitable habitat primarily matching the current distribution. In contrast, models using North American and European records predicted patchier and broader distributions, with additional suitable habitat both northwards and southwards. Niche overlap was accordingly greatest between the models created using North American and European observations (*D = 0.759*) and low for both the North America‐Australia and Europe‐Australia comparison (*D = 0.270* and *D = 0.243*, respectively). Only the *D* estimate for North America‐Europe was significantly higher than expected, with *D* estimates for both North America‐Australia and Europe‐Australia falling within the null distribution (Figure S3). This suggests niche conservation between North American and European populations and supports our initial assumption that the Australian population is not currently in equilibrium.

**FIGURE 2:**
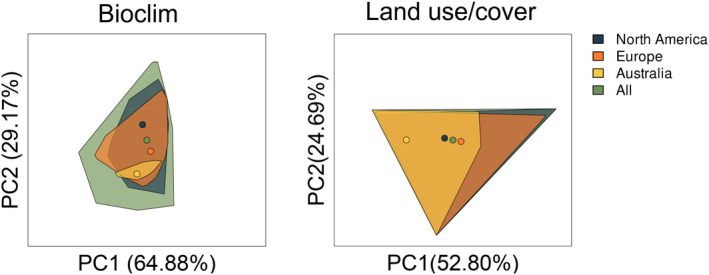
Principal component analysis of 19 bioclimatic and 8 land use/cover variables explaining the distribution of ragweed. For each range, a convex hull was created based on the environmental conditions at records obtained from GBIF as mapped along the first two principal components (PC1 and PC2). The size of the convex hull was used as a proxy for the breadth of the realized niche. The ‘All’ range includes all observations, including those from outside North America, Europe, and Australia. Circles indicate the niche centroid of each range.

With respect to the first two principal components, Australian ragweed occupied a much narrower bioclimatic niche than other populations (Figure 2). Taking the area of the convex hulls as proxies for niche breadth, the Australian niche is only 5.14% of the global bioclimatic niche, a fraction of the North American (51.46% of global niche) or European niche (44.21% of global niche).

Similar results were obtained when considering land use/cover variables as predictors. As before and relative to the global realized niche, ragweed in Australia had a narrower land use/cover niche (64.97% of global niche) than in either Europe (91.47% of global niche) or North America (94.68% of global niche). However, the niche defined by land use/cover was more conserved across ranges than the one defined by bioclimatic variables, with over 90% of the global niche being occupied in Europe and North America.

### 3.2 Genetic structure analysis

**FIGURE 3:**
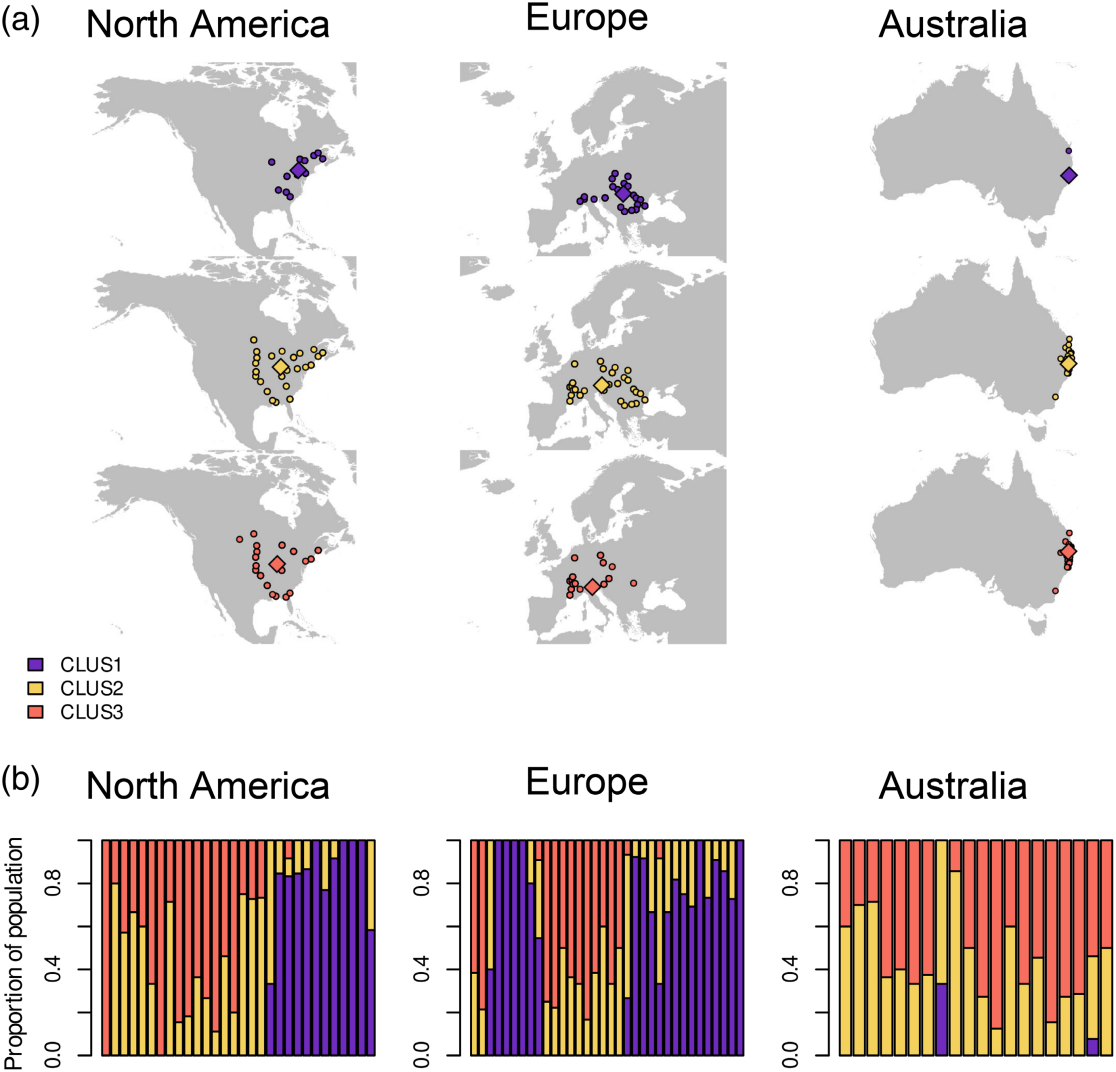
(a) Occurrence records of the different genetic clusters across the three ranges of ragweed. Circles indicate actual occurrences while diamonds indicate the geographic centroid of each cluster. Clustering was performed on admixture proportions assuming three ancestral populations. (b) Distribution of cluster memberships within each population. Each bar represents a population from the given range, with colours indicating the proportion of genotyped individuals belonging to one of three genetic clusters.

The genetic clusters were unevenly distributed across the three ranges (Figure 3a). While all three clusters were common in North America (107 individuals in CLUS1, 101 in CLUS2, 95 in CLUS3) and Europe (176 individuals in CLUS1, 106 in CLUS2, 92 in CLUS3), only 2 out of 183 Australian individuals belonged to CLUS1. Within each population, cluster representation was also uneven: only 5 out of 84 populations contained individuals from all 3 clusters (1 in North America, 3 in Europe, 1 in Australia). Eleven populations only contained individuals from a single cluster: five in North America (excluding one population represented by a single individual) and six in Europe. These genetically homogeneous populations consisted of either CLUS1 or CLUS3 individuals, but never CLUS2 (Figure 3b). Cluster centroids maintained their relative positions in all three ranges, with CLUS2 always located between CLUS1 and CLUS3. Accordingly, CLUS2 is the most widely distributed and found in all populations that were not monotypic.

In addition to its rarity in Australia, CLUS1 is also an outlier with regard to its realized niche. CLUS1 plants (mean annual temperature BIO1 = 9.404°C, annual precipitation BIO12 = 847.600 mm) were generally found in cooler, drier conditions than CLUS2 (BIO1 = 12.111°C, BIO12 = 978.122 mm) and CLUS3 (BIO1 = 13.177°C, BIO12 = 1043.253 mm; see Table S6 for comparisons of all bioclimatic variables). CLUS1 plants also had less overlap with CLUS2 (*D = 0.437*) and CLUS3 (*D = 0.373*) when compared to the overlap between CLUS2 and CLUS3 (*D =* 0.841). This general trend was conserved within each range (Figure S4) and also seen in F_ST_ estimates (Table 1), although whether CLUS1 was more similar with CLUS2 or CLUS3 varied across ranges (Table S7). Moreover, we found no statistically significant relationship between Schoener's *D* and F_ST_ (Table S8) values overall (Mantel test, *r = − 0.0116*, *p*‐value = 0.478).

**TABLE 1:** F_ST_ estimate for different clusters within each range.

Range	CLUS1‐CLUS2	CLUS1‐CLUS3	CLUS2‐CLUS3
Global	0.00378	0.00842	0.00165
North America	0.00526	0.00953	0.00142
Europe	0.00261	0.00942	0.00233
Australia	0.0145	0.0140	0.000252

### 3.3 Distribution of cluster memberships within population and predicted ragweed distribution under different invasion scenarios

**FIGURE 4:**
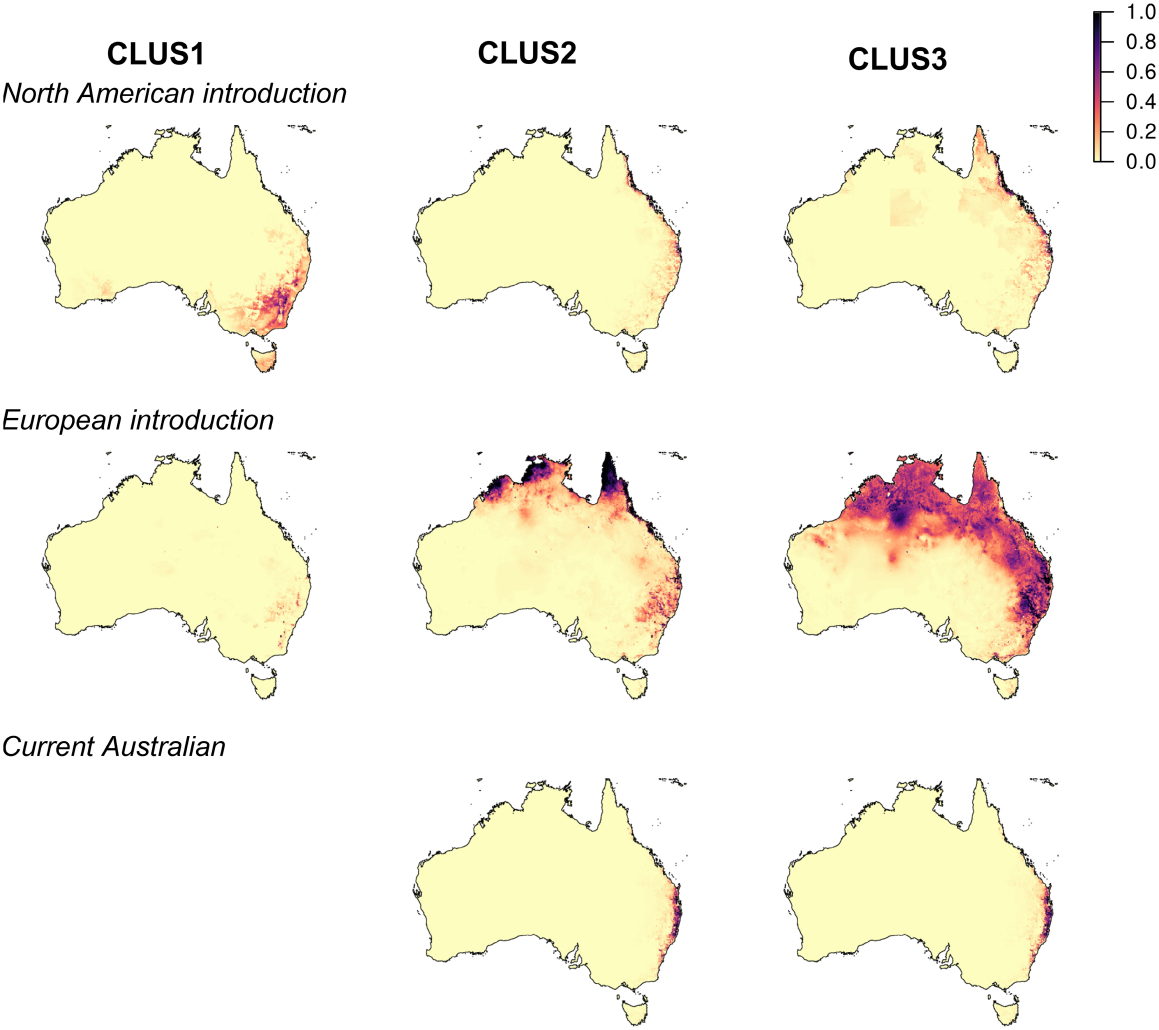
Predicted distribution of *Ambrosia artemisiifolia* under different invasion scenarios involving the introduction of a single cluster from different ranges to Australia. For each scenario, MaxEnt models were created using records of plants assigned to 1 of the 3 clusters as determined through k‐means clustering of ADMIXTURE proportions, 8 land use/cover data, and 19 bioclimatic variables. The three scenarios correspond to the introduction of novel genotypes from North America, novel genotypes from Europe, or current genotypes from Australia without additional genetic variation. The prediction for CLUS1 under the ‘Current Australian’ scenario was excluded due to containing only 2 observations.

Both genetic cluster identity and range of origin affected estimates of the potential distribution of ragweed in Australia (Figure 4). The largest range expansion was predicted for the introduction of CLUS3 plants from Europe (0.278% increase in suitable habitat across the entire continent of Australia), representing a 53% increase relative to the expansion predicted for CLUS2 plants from the same range of origin (0.182% increase). In both instances, habitat suitability was predicted to increase northwards and inland. In contrast, the introduction of CLUS1 plants from North America led to a potential range expansion further south (0.137% increase).

As for the actual model predictions, the extent of model extrapolation depended strongly on genetic cluster identity and range of origin. Extrapolation also varied according to the group of predictors being considered, with greater extrapolation for bioclimatic than land use/cover variables (Figures S5 and S6). Bioclimatic extrapolation was most notable in northern Australia, a region predicted to have high suitability by multiple ENMs but whose values fell outside the observed range for most bioclimatic variables. This contrasted the pattern of extrapolation for land use/cover, where extrapolation was most prominent in central Australia, a region predicted to have low suitability for ragweed.

## 4 DISCUSSION

The genetic composition of source populations is likely to have a profound effect on invasion success and subsequent range expansion. Identifying the most threatening sources of invaders provides valuable information for guiding management practices (Buckley & Catford, 2016), as it allows strategies to be more specific and targeted. For example, the Australian government implemented the Northern Australia Quarantine Strategy (NAQS) to specifically monitor and prevent the entry of pests and diseases through Australia's northern coastline (Anderson et al., 2017). The principles underlying NAQS can be applied to management of common ragweed, as the species shows variable invasive potential due to differences in habitat preference between genetically differentiated clusters. The cluster that occurred in cooler, drier conditions (CLUS1) had a narrower predicted distribution that those found in relatively warmer, wetter locations (CLUS2 and CLUS3). This suggests the introduction of appropriate genetic variation has the potential to greatly increase invasive potential.

### 4.1 Habitat preference is different across ranges

Species can exhibit significant lag times between introduction and spread (Crooks, 2005). Owing to this lag, many invaders occupy only a subset of their potential introduced range. This potential range may also change following additional introductions due to the supplemental introduction of genetic variation or the bypassing of dispersal barriers. Understanding the gap between the potential range of already‐present individuals and those that may be introduced in the future requires the selection of appropriate records during model construction (Kramer‐Schadt et al., 2013; Proosdij et al., 2016).

We first investigated the effect of range of origin on predicted distribution by creating ENMs using range‐segregated records from GBIF.org. These range‐specific ENMs indicate realized niches are differentiated between continents, but the pattern of differentiation depended on the predictors being considered. The land use/cover niche was highly conserved, suggesting it imposes a strong constraint on ragweed distribution. This is consistent with common ragweed's ruderal nature, which necessitates open or disturbed habitats and means the species is often absent from later successions (Gentili et al., 2017). In contrast, the bioclimatic niche was more differentiated between ranges (Figure 2). We observed both niche unfilling and niche expansion in Europe, with plants occurring in climatic conditions not seen in North America. Australian ragweed showed only niche unfilling: their realized niche was much narrower and represented a subset of the European and North American niches. This was expected – niche expansion in Europe compared to North America was previously reported (Sun et al., 2017), while the narrow niche in Australia is reflective of its restricted distribution.

Differences in realized niche size translated to variation in predicted distribution but not model accuracy. Niche unfilling in Australia resulted in high AUC for all ENMs projected to the region, since no Australian ragweed samples were found in a region that was novel with respect to either bioclimatic or land use/cover predictors. Models varied in their predicted habitat suitability outside known occurrence sites, but this does not affect AUC. Between‐range comparisons of *D* were only statistically significant when comparing Europe and North America. For both the Europe‐Australia and North America‐Australia comparisons, we could not distinguish true niche overlap from differences in habitat availability between the ranges. Although this does not necessarily indicate an absence of niche differentiation (Warren et al., 2008), the fact that comparisons including Australia were not significantly different suggests accounting for geographic origin alone is insufficient for detecting niche differentiation if one of the ranges considered is not at equilibrium.

### 4.2 Patterns of genetic diversity in Australian ragweed

Given the importance of environmental factors as a driver of adaptation and genetic differentiation in plants (Anderson & Song, 2020; Lee, 2002), we hypothesized that genetic variation would be correlated with habitat preference. This would, by extension, cause genetic variation to underlie patterns of niche differentiation. Although the actual relationship was not as simple as initially assumed, a clear signal still recurred throughout our data set: CLUS2 and CLUS3 were always the least differentiated and had the greatest niche overlap. This pattern of niche overlap was maintained even in Australia, where prior demographic analysis of genetic data supports a single introduction of admixed European populations (van Boheemen et al., 2017). Assuming this introduction event introduced plants from all three clusters, we would have expected the CLUS3 individuals to remain rare in Australia, reflecting the fact that CLUS3 was always the rarest among European populations carrying individuals from all three clusters (Figure 3). Furthermore, the unpredictability of introduction events should have disrupted the relationship between cluster identity and habitat preference. None of our results reflected these expectations: CLUS1 was rare in Australia instead of CLUS3, and the pattern of niche overlap among clusters remained consistent.

Although we cannot discard the possibility that CLUS1 individuals were actually introduced to Australia at low frequencies and were lost post‐introduction due to genetic drift, another potential explanation for these observations is the strong selection against CLUS1 individuals due to initial maladaptation. CLUS1 plants prefer cooler and drier climates than CLUS2 or CLUS3 plants and are less suited to subtropical Queensland. At the same time, environmental selection pressures similar to those experienced in Europe and North America may have recreated the relationship between genetic and environmental variation established in the other two ranges. Previous studies suggest increased genetic diversity can improve invasion success by providing the standing variation for selection to act upon (Crawford & Whitney, 2010; Hahn & Rieseberg, 2017; Lavergne & Molofsky, 2007). Although our findings do not dispute these results, it does suggest genetic variation that does not support establishment or adaptation may be rapidly lost after introduction (Jacquemyn et al., 2009). These results are consistent with findings in European ragweed, where putatively adaptive loci show evidence of rapid temporal changes (Battlay et al., 2023).

### 4.3 The potential distribution of ragweed in Australia and model caveats

The potential for ragweed range expansion varied according to both the genetic variation being supplied and its range of origin. For example, CLUS3 plants from Europe had a broader predicted distribution in Australia than those coming from North America. The effect of range of origin on predicted distribution is surprising, as we initially hypothesized the niche to be conserved even across ranges due to the overall rarity of niche shifts among invasive plants (Petitpierre et al., 2012). However, these results may be explained by the correlative nature of ecological niche models. Our reliance on occurrence records meant our models could neither capture the fundamental niche nor account for demographic history. The broad realized niche of CLUS3 individuals from Europe could be explained by multiple introductions of ragweed from North America to various locations across Europe (Afonin et al., 2018; Essl et al., 2015; van Boheemen et al., 2017). This would have allowed European ragweed to bypass dispersal barriers and access novel habitats. These novel habitats lacked existing ragweed populations or specialist enemies (Lakeman‐Fraser & Ewers, 2013) that would have prevented incoming individuals from establishing. This contrasts with conditions in its native North American range, where human‐mediated dispersal would have less impact because established ragweed populations would have had their distribution shaped by natural dispersal already.

Another consequence of the reliance on occurrence records is the need to extrapolate models to wholly novel conditions in order to generate predictions. The issue of model extrapolation is exacerbated because genomic clustering shrinks the number of available observations (only a fraction of plant records were genotyped) and the range of environments observed in each model due to genotype‐environment associations. This extensive extrapolation produces models that are less powerful individually due to separating intraspecific niche differentiation into multiple separate models. However, predictions into regions of high uncertainty can be validated using occurrence records without genotype data and considering the biology of the species investigated. For example, our prediction of ragweed occurrence in northern Australia relied on extensive bioclimatic extrapolation but is ultimately reasonable given the presence of ragweed at comparable and lower absolute latitudes (e.g. in Florida, Hawaii, Mexico, and Colombia).

## 5 CONCLUSIONS AND FUTURE DIRECTIONS

Genetic variation present in founding populations is typically considered *a posteriori*, with genetic bottleneck seen as the initial constraint that was successfully overcome by invaders (Lee, 2002) and the remaining genetic variation as the reservoir of adaptive potential that enabled contemporary invasion and further range expansion (Dlugosch & Parker, 2008). Here, we shift perspectives to highlight the importance of considering genetic variation at the early stages of invasion to assess spread risk. Using common ragweed in Australia as a case study, we show how ragweed range expansion on the continent will be contingent on the introduction of specific genotypes that could colonize novel habitats.

Perhaps unsurprisingly, we found the effect of genetic variation itself to be variable. In one case (CLUS1 in Australia), we even found evidence suggesting maladapted genotypes can become rare. Although these findings matched expectations from invasion theory, there are few examples demonstrating the importance of specific genotypes for invasion success. A recent study on another Australian invasive species reported similar results to our work: Alves et al. (2022) found the spread of rabbits in Australia to only occur following the importation and release of more invasive genotypes in 1859, despite the presence of domestic rabbits on the continent since 1788. In another example, the range of an invasive parasite was found to be shaped by the genotype of its host (Mathieu‐Bégné et al., 2021).

An ongoing challenge in invasion biology is to predict invasions before they occur. Our study provides key developments on this front: by incorporating genomic data into the established and commonly used ENM framework, we show how managers can identify the most concerning sources of an invasive species. This information will allow them to implement strategies like increasing the screening of goods coming from specific regions (Buddenhagen et al., 2023) or preventing imports from areas containing genetic variation of high concern.

## FUNDING INFORMATION

This research was funded by the Melbourne Research Scholarship awarded to ARP and the ARC Discovery Project (DP220102362) awarded to KAH and AFL.

## CONFLICT OF INTEREST STATEMENT

The authors declare no conflicts of interest.

## Supporting information


Appendix S1.

## Data Availability

Data sharing is not applicable to this article as no new data were created or analysed in this study.

## References

[eva13632-bib-0001] Afonin, A. N. , Luneva, N. N. , Fedorova, Y. A. , Kletchkovskiy, Y. E. , & Chebanovskaya, A. F. (2018). History of introduction and distribution of common ragweed (*Ambrosia artemisiifolia* L.) in the European part of the Russian Federation and in the Ukraine. EPPO Bulletin, 48(2), 266–273. 10.1111/epp.12484

[eva13632-bib-0002] Ahlroth, P. , Alatalo, R. V. , Holopainen, A. , Kumpulainen, T. , & Suhonen, J. (2003). Founder population size and number of source populations enhance colonization success in waterstriders. Oecologia, 137(4), 617–620. 10.1007/s00442-003-1344-y 14534781

[eva13632-bib-0003] Alexander, D. H. , & Lange, K. (2011). Enhancements to the ADMIXTURE algorithm for individual ancestry estimation. BMC Bioinformatics, 12(1), 246. 10.1186/1471-2105-12-246 21682921 PMC3146885

[eva13632-bib-0107] Alves, J. M. , Carneiro, M. , Day, J. P. , Welch, J. J. , Duckworth, J. A. , Cox, T. E. , Letnic, M. , Strive, T. , Ferrand, N. , & Jiggins, F. M. (2022). A single introduction of wild rabbits triggered the biological invasion of Australia. Proceedings of the National Academy of Sciences of the United States of America, 119(35), e2122734119. 10.1073/pnas.2122734119 35994668 PMC9436340

[eva13632-bib-0004] Anderson, C. , Low‐Choy, S. , Whittle, P. , Taylor, S. , Gambley, C. , Smith, L. , Gillespie, P. , Löcker, H. , Davis, R. , & Dominiak, B. (2017). Australian plant biosecurity surveillance systems. Crop Protection, 100, 8–20. 10.1016/j.cropro.2017.05.023

[eva13632-bib-0005] Anderson, J. T. , & Song, B.‐H. (2020). Plant adaptation to climate change—Where are we? Journal of Systematics and Evolution, 58(5), 533–545. 10.1111/jse.12649 33584833 PMC7875155

[eva13632-bib-0006] Atlas of Living Australia . (2023). Website list . https://lists.ala.org.au/speciesListItem/list/dr823

[eva13632-bib-0007] Atwater, D. Z. , Ervine, C. , & Barney, J. N. (2018). Climatic niche shifts are common in introduced plants. Nature Ecology & Evolution, 2(1), 34–43. 10.1038/s41559-017-0396-z 29203919

[eva13632-bib-0008] Barbet‐Massin, M. , Rome, Q. , Villemant, C. , & Courchamp, F. (2018). Can species distribution models really predict the expansion of invasive species? PLoS One, 13(3), e0193085. 10.1371/journal.pone.0193085 29509789 PMC5839551

[eva13632-bib-0009] Bass, D. J. , Delpech, V. , Beard, J. , Bass, P. , & Walls, R. S. (2000). Ragweed in Australia. Aerobiologia, 16(1), 107–111. 10.1023/A:1007696112953

[eva13632-bib-0010] Battlay, P. , Wilson, J. , Bieker, V. C. , Lee, C. , Prapas, D. , Petersen, B. , Craig, S. , van Boheemen, L. , Scalone, R. , de Silva, N. P. , Sharma, A. , Konstantinović, B. , Nurkowski, K. A. , Rieseberg, L. H. , Connallon, T. , Martin, M. D. , & Hodgins, K. A. (2023). Large haploblocks underlie rapid adaptation in the invasive weed *Ambrosia artemisiifolia* . Nature Communications, 14(1), 1717. 10.1038/s41467-023-37303-4 PMC10042993 36973251

[eva13632-bib-0011] Bhattarai, G. P. , Meyerson, L. A. , Anderson, J. , Cummings, D. , Allen, W. J. , & Cronin, J. T. (2017). Biogeography of a plant invasion: Genetic variation and plasticity in latitudinal clines for traits related to herbivory. Ecological Monographs, 87(1), 57–75. 10.1002/ecm.1233

[eva13632-bib-0012] Bieker, V. C. , Battlay, P. , Petersen, B. , Sun, X. , Wilson, J. , Brealey, J. C. , Bretagnolle, F. , Nurkowski, K. , Lee, C. , Barreiro, F. S. , Owens, G. L. , Lee, J. Y. , Kellner, F. L. , van Boheeman, L. , Gopalakrishnan, S. , Gaudeul, M. , Mueller‐Schaerer, H. , Lommen, S. , Karrer, G. , … Martin, M. D. (2022). Uncovering the genomic basis of an extraordinary plant invasion. Science Advances, 8(34), eabo5115. 10.1126/sciadv.abo5115 36001672 PMC9401624

[eva13632-bib-0013] Blackburn, T. M. , Lockwood, J. L. , & Cassey, P. (2015). The influence of numbers on invasion success. Molecular Ecology, 24(9), 1942–1953. 10.1111/mec.13075 25641210

[eva13632-bib-0014] Blossey, B. (1999). Before, during and after: The need for long‐term monitoring in invasive plant species management. Biological Invasions, 1(2), 301–311. 10.1023/A:1010084724526

[eva13632-bib-0015] Bonnamour, A. , Gippet, J. M. W. , & Bertelsmeier, C. (2021). Insect and plant invasions follow two waves of globalisation. Ecology Letters, 24(11), 2418–2426. 10.1111/ele.13863 34420251 PMC9290749

[eva13632-bib-0016] Briscoe Runquist, R. D. , Lake, T. , Tiffin, P. , & Moeller, D. A. (2019). Species distribution models throughout the invasion history of Palmer amaranth predict regions at risk of future invasion and reveal challenges with modeling rapidly shifting geographic ranges. Scientific Reports, 9(1), 2426. 10.1038/s41598-018-38054-9 30787301 PMC6382853

[eva13632-bib-0017] Broennimann, O. , Treier, U. A. , Müller‐Schärer, H. , Thuiller, W. , Peterson, A. T. , & Guisan, A. (2007). Evidence of climatic niche shift during biological invasion. Ecology Letters, 10(8), 701–709. 10.1111/j.1461-0248.2007.01060.x 17594425

[eva13632-bib-0018] Browning, B. L. , Zhou, Y. , & Browning, S. R. (2018). A one‐penny imputed genome from next‐generation reference panels. American Journal of Human Genetics, 103(3), 338–348. 10.1016/j.ajhg.2018.07.015 30100085 PMC6128308

[eva13632-bib-0019] Buckley, Y. M. , & Catford, J. (2016). Does the biogeographic origin of species matter? Ecological effects of native and non‐native species and the use of origin to guide management. Journal of Ecology, 104(1), 4–17. 10.1111/1365-2745.12501

[eva13632-bib-0020] Buddenhagen, C. E. , Hackell, D. , Henderson, H. V. , & Wynne‐Jones, B. (2023). Factors impacting the detection of weed seed contaminants in seed lots. Pest Management Science, 79(2), 881–890. 10.1002/ps.7257 36308732 PMC10099985

[eva13632-bib-0021] Chapman, D. S. , Makra, L. , Albertini, R. , Bonini, M. , Páldy, A. , Rodinkova, V. , Šikoparija, B. , Weryszko‐Chmielewska, E. , & Bullock, J. M. (2016). Modelling the introduction and spread of non‐native species: International trade and climate change drive ragweed invasion. Global Change Biology, 22(9), 3067–3079. 10.1111/gcb.13220 26748862

[eva13632-bib-0022] Chapman, D. S. , Scalone, R. , Štefanić, E. , & Bullock, J. M. (2017). Mechanistic species distribution modeling reveals a niche shift during invasion. Ecology, 98(6), 1671–1680. 10.1002/ecy.1835 28369815

[eva13632-bib-0023] Chikoye, D. , Weise, S. F. , & Swanton, C. J. (1995). Influence of common Ragweed (*Ambrosia artemisiifolia*) time of emergence and density on white bean (*Phaseolus vulgaris*). Weed Science, 43(3), 375–380.

[eva13632-bib-0024] Coble, H. D. , Williams, F. M. , & Ritter, R. L. (1981). Common Ragweed (*Ambrosia artemisiifolia*) interference in soybeans (*Glycine max*). Weed Science, 29(3), 339–342. 10.1017/S0043174500062081

[eva13632-bib-0025] Cobos, M. E. , Owens, H. L. , Soberón, J. , & Townsend, P. A. (2023). mop: Mobility oriented‐parity metric . R package version 0.1.1. https://CRAN.R‐project.org/package=mop

[eva13632-bib-0026] Crawford, K. M. , & Whitney, K. D. (2010). Population genetic diversity influences colonization success. Molecular Ecology, 19(6), 1253–1263. 10.1111/j.1365-294X.2010.04550.x 20456227

[eva13632-bib-0027] Crooks, J. A. (2005). Lag times and exotic species: The ecology and management of biological invasions in slow‐motion1. Écoscience, 12(3), 316–329. 10.2980/i1195-6860-12-3-316.1

[eva13632-bib-0029] Dahl, Å. , Strandhede, S.‐O. , & Wihl, J.‐Å. (1999). Ragweed – An allergy risk in Sweden? Aerobiologia, 15(4), 293–297. 10.1023/A:1007678107552

[eva13632-bib-0030] Danecek, P. , Auton, A. , Abecasis, G. , Albers, C. A. , Banks, E. , DePristo, M. A. , Handsaker, R. E. , Lunter, G. , Marth, G. T. , Sherry, S. T. , McVean, G. , Durbin, R. , & 1000 Genomes Project Analysis Group . (2011). The variant call format and VCFtools. Bioinformatics, 27(15), 2156–2158. 10.1093/bioinformatics/btr330 21653522 PMC3137218

[eva13632-bib-0031] Dlugosch, K. M. , & Parker, I. M. (2008). Founding events in species invasions: Genetic variation, adaptive evolution, and the role of multiple introductions. Molecular Ecology, 17(1), 431–449. 10.1111/j.1365-294X.2007.03538.x 17908213

[eva13632-bib-0032] Essl, F. , Biró, K. , Brandes, D. , Broennimann, O. , Bullock, J. M. , Chapman, D. S. , Chauvel, B. , Dullinger, S. , Fumanal, B. , Guisan, A. , Karrer, G. , Kazinczi, G. , Kueffer, C. , Laitung, B. , Lavoie, C. , Leitner, M. , Mang, T. , Moser, D. , Müller‐Schärer, H. , … Follak, S. (2015). Biological flora of the British Isles: *Ambrosia artemisiifolia* . Journal of Ecology, 103(4), 1069–1098. 10.1111/1365-2745.12424

[eva13632-bib-0106] Evanno, G. , Regnau, S. , & Goudet, J. (2005). Detecting the number of clusters of individuals using the software STRUCTURE: A simulation study. Molecular Ecology, 14(8), 2611–2620. 10.1111/j.1365-294X.2005.02553.x. 15969739

[eva13632-bib-0033] Fick, S. E. , & Hijmans, R. J. (2017). WorldClim 2: New 1‐km spatial resolution climate surfaces for global land areas. International Journal of Climatology, 37(12), 4302–4315. 10.1002/joc.5086

[eva13632-bib-0034] Fischer, G. , Nachtergaele, F. , Prieler, S. , van Velthuizen, H. T. , Verelst, L. , & Wiberg, D. (2008). Global agro‐ecological zones assessment for agriculture (GAEZ 2008) (p. 10). IIASA.

[eva13632-bib-0035] Flory, S. L. , & Clay, K. (2013). Pathogen accumulation and long‐term dynamics of plant invasions. Journal of Ecology, 101, 607–613.

[eva13632-bib-0036] Fournier‐Level, A. , Korte, A. , Cooper, M. D. , Nordborg, M. , Schmitt, J. , & Wilczek, A. M. (2011). A map of local adaptation in *Arabidopsis thaliana* . Science, 334(6052), 86–89. 10.1126/science.1209271 21980109

[eva13632-bib-0037] GBIF Secretariat . (2023). Ambrosia artemisiifolia L. in GBIF Secretariat. GBIF backbone taxonomy. Checklist dataset . 10.15468/39omei. accessed via GBIF.org

[eva13632-bib-0038] GBIF.org . (2023). GBIF occurrence . 10.15468/dl.bw8yx8

[eva13632-bib-0039] Gentili, R. , Montagnani, C. , Gilardelli, F. , Guarino, M. F. , & Citterio, S. (2017). Let native species take their course: *Ambrosia artemisiifolia* replacement during natural or “artificial” succession. Acta Oecologica, 82, 32–40. 10.1016/j.actao.2017.05.007

[eva13632-bib-0040] Grant, A.‐G. , & Kalisz, S. (2020). Do selfing species have greater niche breadth? Support from ecological niche modeling. Evolution, 74(1), 73–88. 10.1111/evo.13870 31707744

[eva13632-bib-0041] Guillera‐Arroita, G. , Lahoz‐Monfort, J. J. , Elith, J. , Gordon, A. , Kujala, H. , Lentini, P. E. , McCarthy, M. A. , Tingley, R. , & Wintle, B. A. (2015). Is my species distribution model fit for purpose? Matching data and models to applications. Global Ecology and Biogeography, 24(3), 276–292. 10.1111/geb.12268

[eva13632-bib-0042] Guisan, A. , & Thuiller, W. (2005). Predicting species distribution: Offering more than simple habitat models. Ecology Letters, 8(9), 993–1009. 10.1111/j.1461-0248.2005.00792.x 34517687

[eva13632-bib-0043] Gurevitch, J. , & Padilla, D. K. (2004). Are invasive species a major cause of extinctions? Trends in Ecology & Evolution, 19(9), 470–474. 10.1016/j.tree.2004.07.005 16701309

[eva13632-bib-0044] Hahn, M. A. , & Rieseberg, L. H. (2017). Genetic admixture and heterosis may enhance the invasiveness of common ragweed. Evolutionary Applications, 10(3), 241–250. 10.1111/eva.12445 28250809 PMC5322403

[eva13632-bib-0045] Hällfors, M. H. , Liao, J. , Dzurisin, J. , Grundel, R. , Hyvärinen, M. , Towle, K. , Wu, G. C. , & Hellmann, J. J. (2016). Addressing potential local adaptation in species distribution models: Implications for conservation under climate change. Ecological Applications, 26(4), 1154–1169. 10.1890/15-0926 27509755

[eva13632-bib-0046] Hijmans, R. J. (2023). predicts: Spatial prediction tools . R package version 0.1–8. https://CRAN.R‐project.org/package=predicts

[eva13632-bib-0047] Hijmans, R. J. , Phillips, S. , Leathwick, J. , & Elith, J. (2021). dismo: Species distribution modeling (1.3‐5) . https://CRAN.R‐project.org/package=dismo

[eva13632-bib-0048] Hijmans, R. J. , van Etten, J. , Sumner, M. , Cheng, J. , Baston, D. , Bevan, A. , Bivand, R. , Busetto, L. , Canty, M. , Forrest, D. , Ghosh, A. , Golicher, D. , Gray, J. , Greenberg, J. A. , Hiemstra, P. , Hingee, K. , Institute for Mathematics Applied Geosciences , Karney, C. , Mattiuzzi, M. , … Wueest, R. (2020). *raster: Geographic data analysis and modeling* (3.3–13). https://CRAN.R‐project.org/package=raster

[eva13632-bib-0050] Hu, Z.‐M. , Zhang, Q.‐S. , Zhang, J. , Kass, J. M. , Mammola, S. , Fresia, P. , Draisma, S. G. A. , Assis, J. , Jueterbock, A. , Yokota, M. , & Zhang, Z. (2021). Intraspecific genetic variation matters when predicting seagrass distribution under climate change. Molecular Ecology, 30(15), 3840–3855. 10.1111/mec.15996 34022079

[eva13632-bib-0051] Hulme, P. E. (2009). Trade, transport and trouble: Managing invasive species pathways in an era of globalization. Journal of Applied Ecology, 46(1), 10–18. 10.1111/j.1365-2664.2008.01600.x

[eva13632-bib-0052] Jacquemyn, H. , Vandepitte, K. , Roldán‐Ruiz, I. , & Honnay, O. (2009). Rapid loss of genetic variation in a founding population of *Primula elatior* (Primulaceae) after colonization. Annals of Botany, 103(5), 777–783. 10.1093/aob/mcn253 19106180 PMC2707865

[eva13632-bib-0053] Katz, D. S. W. , Connor Barrie, B. T. , & Carey, T. S. (2014). Urban ragweed populations in vacant lots: An ecological perspective on management. Urban Forestry & Urban Greening, 13(4), 756–760. 10.1016/j.ufug.2014.06.001

[eva13632-bib-0054] Keller, R. P. , Frang, K. , & Lodge, D. M. (2008). Preventing the spread of invasive species: Economic benefits of intervention guided by ecological predictions. Conservation Biology, 22(1), 80–88. 10.1111/j.1523-1739.2007.00811.x 18254855

[eva13632-bib-0055] Kramer‐Schadt, S. , Niedballa, J. , Pilgrim, J. D. , Schröder, B. , Lindenborn, J. , Reinfelder, V. , Stillfried, M. , Heckmann, I. , Scharf, A. K. , Augeri, D. M. , Cheyne, S. M. , Hearn, A. J. , Ross, J. , Macdonald, D. W. , Mathai, J. , Eaton, J. , Marshall, A. J. , Semiadi, G. , Rustam, R. , … Wilting, A. (2013). The importance of correcting for sampling bias in MaxEnt species distribution models. Diversity and Distributions, 19(11), 1366–1379. 10.1111/ddi.12096

[eva13632-bib-0056] Kumar Rai, P. , & Singh, J. S. (2020). Invasive alien plant species: Their impact on environment, ecosystem services and human health. Ecological Indicators, 111, 106020. 10.1016/j.ecolind.2019.106020 32372880 PMC7194640

[eva13632-bib-0057] Laaidi, M. , Laaidi, K. , Besancenot, J.‐P. , & Thibaudon, M. (2003). Ragweed in France: An invasive plant and its allergenic pollen. Annals of Allergy, Asthma & Immunology, 91(2), 195–201. 10.1016/S1081-1206(10)62177-1 12952115

[eva13632-bib-0058] Lakeman‐Fraser, P. , & Ewers, R. M. (2013). Enemy release promotes range expansion in a host plant. Oecologia, 172(4), 1203–1212. 10.1007/s00442-012-2555-x 23239216

[eva13632-bib-0059] Lavergne, S. , & Molofsky, J. (2007). Increased genetic variation and evolutionary potential drive the success of an invasive grass. Proceedings of the National Academy of Sciences of the United States of America, 104(10), 3883–3888. 10.1073/pnas.0607324104 17360447 PMC1805698

[eva13632-bib-0060] Lecocq, T. , Rasmont, P. , Harpke, A. , & Schweiger, O. (2016). Improving international trade regulation by considering intraspecific variation for invasion risk assessment of commercially traded species: The *Bombus terrestris* case. Conservation Letters, 9(4), 281–289. 10.1111/conl.12215

[eva13632-bib-0061] Lee, C. E. (2002). Evolutionary genetics of invasive species. Trends in Ecology & Evolution, 17(8), 386–391. 10.1016/S0169-5347(02)02554-5

[eva13632-bib-0062] Lemke, A. , Buchholz, S. , Kowarik, I. , Starfinger, U. , & von der Lippe, M. (2021). Interaction of traffic intensity and habitat features shape invasion dynamics of an invasive alien species (*Ambrosia artemisiifolia*) in a regional road network. NeoBiota, 64, 155–175. 10.3897/neobiota.64.58775

[eva13632-bib-0063] Leung, B. , Lodge, D. M. , Finnoff, D. , Shogren, J. F. , Lewis, M. A. , & Lamberti, G. (2002). An ounce of prevention or a pound of cure: Bioeconomic risk analysis of invasive species. Proceedings of the Royal Society of London, Series B: Biological Sciences, 269(1508), 2407–2413. 10.1098/rspb.2002.2179 PMC1691180 12495482

[eva13632-bib-0064] Li, H. (2013). Aligning sequence reads, clone sequences and assembly contigs with BWA‐MEM . (arXiv:1303.3997). arXiv 10.48550/arXiv.1303.3997

[eva13632-bib-0065] Liu, C. , Wolter, C. , Xian, W. , & Jeschke, J. M. (2020). Species distribution models have limited spatial transferability for invasive species. Ecology Letters, 23(11), 1682–1692. 10.1111/ele.13577 32881373

[eva13632-bib-0066] Lockwood, J. L. , Cassey, P. , & Blackburn, T. M. (2009). The more you introduce the more you get: The role of colonization pressure and propagule pressure in invasion ecology. Diversity and Distributions, 15(5), 904–910. 10.1111/j.1472-4642.2009.00594.x

[eva13632-bib-0067] Martin, M. D. , Zimmer, E. A. , Olsen, M. T. , Foote, A. D. , Gilbert, M. T. P. , & Brush, G. S. (2014). Herbarium specimens reveal a historical shift in phylogeographic structure of common ragweed during native range disturbance. Molecular Ecology, 23(7), 1701–1716. 10.1111/mec.12675 24450363

[eva13632-bib-0068] Mathieu‐Bégné, E. , Loot, G. , Mazé‐Guilmo, E. , Mullet, V. , Genthon, C. , & Blanchet, S. (2021). Combining species distribution models and population genomics underlines the determinants of range limitation in an emerging parasite. Ecography, 44(2), 307–319. 10.1111/ecog.05301

[eva13632-bib-0069] Merow, C. , Smith, M. J. , & Silander, J. A. (2013). A practical guide to MaxEnt for modeling species' distributions: What it does, and why inputs and settings matter. Ecography, 36(10), 1058–1069. 10.1111/j.1600-0587.2013.07872.x

[eva13632-bib-0070] Moran, E. V. , & Alexander, J. M. (2014). Evolutionary responses to global change: Lessons from invasive species. Ecology Letters, 17(5), 637–649. 10.1111/ele.12262 24612028

[eva13632-bib-0071] Oswalt, M. L. , & Marshall, G. D. (2008). Ragweed as an example of worldwide allergen expansion. Allergy, Asthma and Clinical Immunology, 4(3), 130–135. 10.1186/1710-1492-4-3-130 PMC2868868 20525135

[eva13632-bib-0072] Owens, H. L. , Campbell, L. P. , Dornak, L. L. , Saupe, E. E. , Barve, N. , Soberón, J. , Ingenloff, K. , Lira‐Noriega, A. , Hensz, C. M. , Myers, C. E. , & Peterson, A. T. (2013). Constraints on interpretation of ecological niche models by limited environmental ranges on calibration areas. Ecological Modelling, 263, 10–18. 10.1016/j.ecolmodel.2013.04.011

[eva13632-bib-0073] Peterson, A. T. (2006). Uses and requirements of ecological niche models and related distributional models. Biodiversity Informatics, 3. 10.17161/bi.v3i0.29

[eva13632-bib-0074] Peterson, A. T. , Stewart, A. , Mohamed, K. I. , & Araújo, M. B. (2008). Shifting global invasive potential of european plants with climate change. PLoS One, 3(6), e2441. 10.1371/journal.pone.0002441 18560572 PMC2409072

[eva13632-bib-0075] Petitpierre, B. , Kueffer, C. , Broennimann, O. , Randin, C. , Daehler, C. , & Guisan, A. (2012). Climatic niche shifts are rare among terrestrial plant invaders. Science, 335(6074), 1344–1348. 10.1126/science.1215933 22422981

[eva13632-bib-0076] Phillips, S. J. , Anderson, R. P. , & Schapire, R. E. (2006). Maximum entropy modeling of species geographic distributions. Ecological Modelling, 190(3), 231–259. 10.1016/j.ecolmodel.2005.03.026

[eva13632-bib-0077] Poplin, R. , Ruano‐Rubio, V. , DePristo, M. A. , Fennell, T. J. , Carneiro, M. O. , der Auwera, G. A. V. , Kling, D. E. , Gauthier, L. D. , Levy‐Moonshine, A. , Roazen, D. , Shakir, K. , Thibault, J. , Chandran, S. , Whelan, C. , Lek, M. , Gabriel, S. , Daly, M. J. , Neale, B. , MacArthur, D. G. , & Banks, E. (2018). Scaling accurate genetic variant discovery to tens of thousands of samples . (p. 201178) 10.1101/201178

[eva13632-bib-0078] van Proosdij, A. S. J. , Sosef, M. S. M. , Wieringa, J. J. , & Raes, N. (2016). Minimum required number of specimen records to develop accurate species distribution models. Ecography, 39(6), 542–552. 10.1111/ecog.01509

[eva13632-bib-0079] Rasmussen, K. , Thyrring, J. , Muscarella, R. , & Borchsenius, F. (2017). Climate‐change‐induced range shifts of three allergenic ragweeds (*Ambrosia* L.) in Europe and their potential impact on human health. PeerJ, 5, e3104. 10.7717/peerj.3104 28321366 PMC5357339

[eva13632-bib-0080] Razgour, O. , Forester, B. , Taggart, J. B. , Bekaert, M. , Juste, J. , Ibáñez, C. , Puechmaille, S. J. , Novella‐Fernandez, R. , Alberdi, A. , & Manel, S. (2019). Considering adaptive genetic variation in climate change vulnerability assessment reduces species range loss projections. Proceedings of the National Academy of Sciences of the United States of America, 116(21), 10418–10423. 10.1073/pnas.1820663116 31061126 PMC6535011

[eva13632-bib-0081] Richardson, D. M. , Pysek, P. , Rejmanek, M. , Barbour, M. G. , Panetta, F. D. , & West, C. J. (2000). Naturalization and invasion of alien plants: Concepts and definitions. Diversity and Distributions, 6(2), 93–107. 10.1046/j.1472-4642.2000.00083.x

[eva13632-bib-0082] Richter, R. , Berger, U. E. , Dullinger, S. , Essl, F. , Leitner, M. , Smith, M. , & Vogl, G. (2013). Spread of invasive ragweed: Climate change, management and how to reduce allergy costs. Journal of Applied Ecology, 50(6), 1422–1430. 10.1111/1365-2664.12156

[eva13632-bib-0083] Rodríguez‐Rodríguez, E. J. , Beltrán, J. F. , Tejedo, M. , Nicieza, A. G. , Llusia, D. , Márquez, R. , & Aragón, P. (2020). Niche models at inter‐ and intraspecific levels reveal hierarchical niche differentiation in midwife toads. Scientific Reports, 10(1), 10942. 10.1038/s41598-020-67992-6 32616878 PMC7331615

[eva13632-bib-0084] Scalone, R. , Lemke, A. , Štefanić, E. , Kolseth, A.‐K. , Rašić, S. , & Andersson, L. (2016). Phenological variation in *Ambrosia artemisiifolia* L. facilitates near future establishment at northern latitudes. PLoS One, 11(11), e0166510. 10.1371/journal.pone.0166510 27846312 PMC5113013

[eva13632-bib-0085] Schaffner, U. , Steinbach, S. , Sun, Y. , Skjøth, C. A. , de Weger, L. A. , Lommen, S. T. , Augustinus, B. A. , Bonini, M. , Karrer, G. , Šikoparija, B. , Thibaudon, M. , & Müller‐Schärer, H. (2020). Biological weed control to relieve millions from *Ambrosia* allergies in Europe. Nature Communications, 11(1), 1745. 10.1038/s41467-020-15586-1 PMC7174423 32317698

[eva13632-bib-0086] Schoener, T. W. (1968). The anolis lizards of Bimini: Resource partitioning in a complex fauna. Ecology, 49(4), 704–726. 10.2307/1935534

[eva13632-bib-0087] Schubert, M. , Ermini, L. , Sarkissian, C. D. , Jónsson, H. , Ginolhac, A. , Schaefer, R. , Martin, M. D. , Fernández, R. , Kircher, M. , McCue, M. , Willerslev, E. , & Orlando, L. (2014). Characterization of ancient and modern genomes by SNP detection and phylogenomic and metagenomic analysis using PALEOMIX. Nature Protocols, 9(5), 1056–1082. 10.1038/nprot.2014.063 24722405

[eva13632-bib-0088] Seebens, H. , Blackburn, T. M. , Dyer, E. E. , Genovesi, P. , Hulme, P. E. , Jeschke, J. M. , Pagad, S. , Pyšek, P. , Winter, M. , Arianoutsou, M. , Bacher, S. , Blasius, B. , Brundu, G. , Capinha, C. , Celesti‐Grapow, L. , Dawson, W. , Dullinger, S. , Fuentes, N. , Jäger, H. , … Essl, F. (2017). No saturation in the accumulation of alien species worldwide. Nature Communications, 8(1), 14435. 10.1038/ncomms14435 PMC5316856 28198420

[eva13632-bib-0089] Sheth, S. N. , Morueta‐Holme, N. , & Angert, A. L. (2020). Determinants of geographic range size in plants. New Phytologist, 226(3), 650–665. 10.1111/nph.16406 31901139

[eva13632-bib-0091] Sun, Y. , Brönnimann, O. , Roderick, G. K. , Poltavsky, A. , Lommen, S. T. E. , & Müller‐Schärer, H. (2017). Climatic suitability ranking of biological control candidates: A biogeographic approach for ragweed management in Europe. Ecosphere, 8(4), e01731. 10.1002/ecs2.1731

[eva13632-bib-0092] Sung, S. , Kwon, Y.‐S. , Lee, D. K. , & Cho, Y. (2018). Predicting the potential distribution of an invasive species, *Solenopsis invicta Buren* (Hymenoptera: Formicidae), under climate change using species distribution models. Entomological Research, 48(6), 505–513. 10.1111/1748-5967.12325

[eva13632-bib-0093] Tischler, G. , & Leonard, S. (2014). biobambam: Tools for read pair collation based algorithms on BAM files. Source Code for Biology and Medicine, 9, 13. 10.1186/1751-0473-9-13

[eva13632-bib-0094] Tobin, P. C. (2018). Managing invasive species. F1000Research, 7. F1000 Faculty Rev‐1686. 10.12688/f1000research.15414.1 PMC6206619 30416712

[eva13632-bib-0095] Václavík, T. , & Meentemeyer, R. K. (2012). Equilibrium or not? Modelling potential distribution of invasive species in different stages of invasion. Diversity and Distributions, 18(1), 73–83. 10.1111/j.1472-4642.2011.00854.x

[eva13632-bib-0096] Valavi, R. , Guillera‐Arroita, G. , Lahoz‐Monfort, J. J. , & Elith, J. (2022). Predictive performance of presence‐only species distribution models: A benchmark study with reproducible code. Ecological Monographs, 92(1), e01486. 10.1002/ecm.1486

[eva13632-bib-0097] van Boheemen, L. A. , Atwater, D. Z. , & Hodgins, K. A. (2019). Rapid and repeated local adaptation to climate in an invasive plant. New Phytologist, 222(1), 614–627. 10.1111/nph.15564 30367474

[eva13632-bib-0098] van Boheemen, L. A. , & Hodgins, K. A. (2020). Rapid repeatable phenotypic and genomic adaptation following multiple introductions. Molecular Ecology, 29(21), 4102–4117. 10.1111/mec.15429 32246535

[eva13632-bib-0099] van Boheemen, L. A. , Lombaert, E. , Nurkowski, K. A. , Gauffre, B. , Rieseberg, L. H. , & Hodgins, K. A. (2017). Multiple introductions, admixture and bridgehead invasion characterize the introduction history of *Ambrosia artemisiifolia* in Europe and Australia. Molecular Ecology, 26(20), 5421–5434. 10.1111/mec.14293 28802079

[eva13632-bib-0100] van Kleunen, M. , Dawson, W. , Essl, F. , Pergl, J. , Winter, M. , Weber, E. , Kreft, H. , Weigelt, P. , Kartesz, J. , Nishino, M. , Antonova, L. A. , Barcelona, J. F. , Cabezas, F. J. , Cárdenas, D. , Cárdenas‐Toro, J. , Castaño, N. , Chacón, E. , Chatelain, C. , Ebel, A. L. , … Pyšek, P. (2015). Global exchange and accumulation of non‐native plants. Nature, 525(7567), 100–103. 10.1038/nature14910 26287466

[eva13632-bib-0101] Warren, D. L. , Glor, R. E. , & Turelli, M. (2008). Environmental niche equivalency versus conservatism: Quantitative approaches to niche evolution. Evolution, 62(11), 2868–2883. 10.1111/j.1558-5646.2008.00482.x 18752605

[eva13632-bib-0102] Wellband, K. W. , Pettitt‐Wade, H. , Fisk, A. T. , & Heath, D. D. (2018). Standing genetic diversity and selection at functional gene loci are associated with differential invasion success in two non‐native fish species. Molecular Ecology, 27(7), 1572–1585. 10.1111/mec.14557 29573310

[eva13632-bib-0103] Willi, Y. , van Buskirk, J. , & Hoffmann, A. A. (2006). Limits to the adaptive potential of small populations. Annual Review of Ecology, Evolution, and Systematics, 37(1), 433–458. 10.1146/annurev.ecolsys.37.091305.110145

[eva13632-bib-0104] Williamson, M. , & Fitter, A. (1996). The varying success of invaders. Ecology, 77(6), 1661–1666. 10.2307/2265769

[eva13632-bib-0105] Zenni, R. D. , & Nuñez, M. A. (2013). The elephant in the room: The role of failed invasions in understanding invasion biology. Oikos, 122(6), 801–815. 10.1111/j.1600-0706.2012.00254.x

