# Supplementary material for: Assessing the invasive potential of different source populations of ragweed (Ambrosia artemisiifolia L.) through genomically informed species distribution modelling
Source: Evol Appl. 2023 Dec 20;17(1):e13632. doi: 10.1111/eva.13632 (PMC10810254; doi:10.1111/eva.13632)
Supplement: Supplementary file 1 — Appendix S1. [file EVA-17-e13632-s001.pdf]

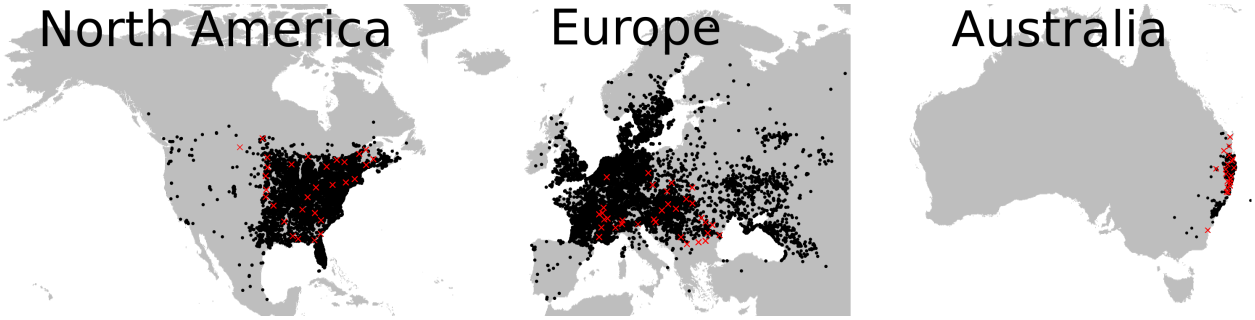

Figure S1. Location of ragweed occurrences on GBIF (black circles) and for the genotyped samples (red crosses) in North America, Europe, and Australia. GBIF occurrences were downsampled to one observation per 2.5’ x 2.5’ cell.

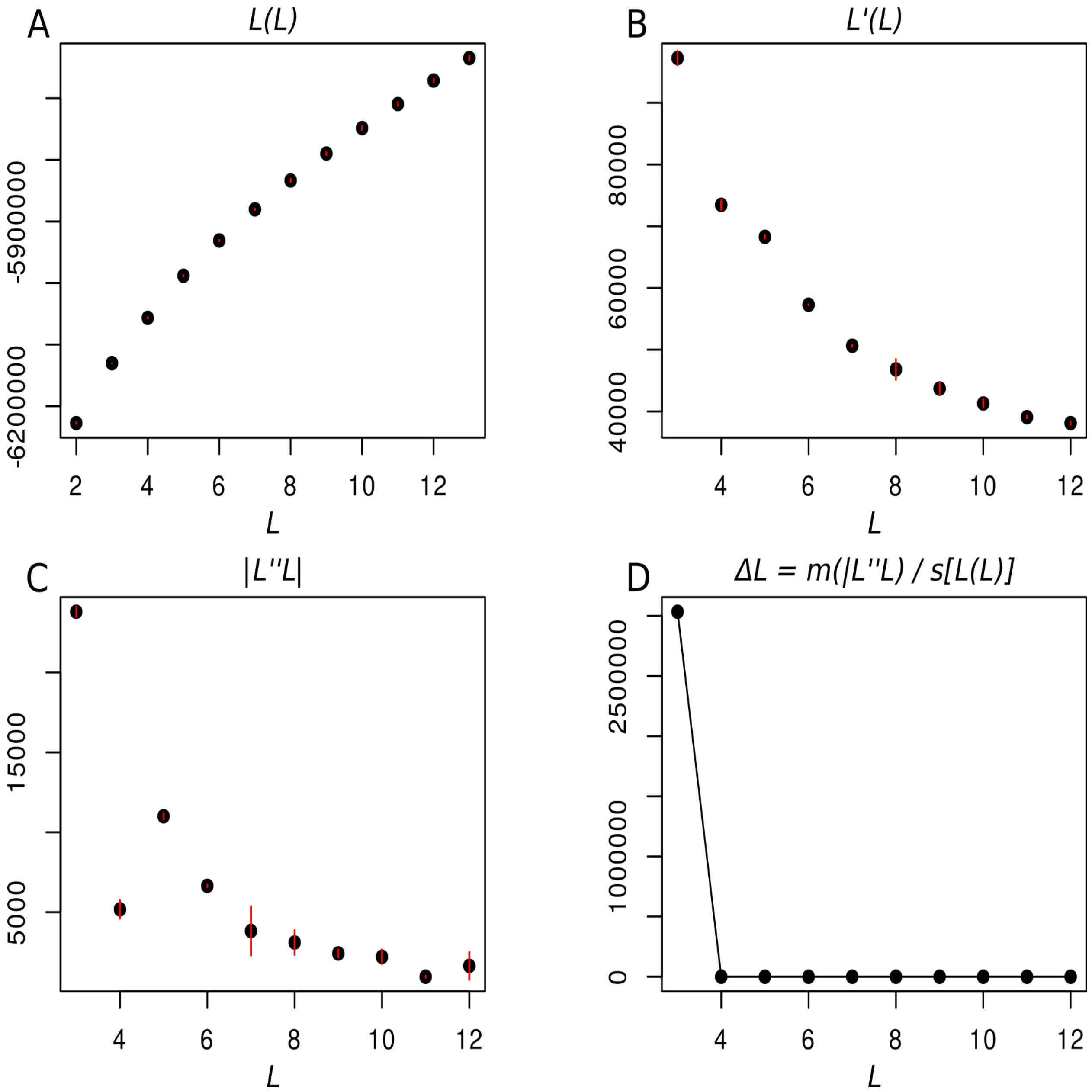

Figure S2. Effect of varying the number of ancestral populations L on model log likelihood. A) Model log likelihood *L(L)*. B) The first order rate of change in log likelihood *L'(L)*. C) The absolute value of the second order rate of change |*L''L*|. D) The mean of the second order rate of change in log likelihood standardised by the standard deviation of the model log likelihoods. Red lines indicate the standard deviation of model log likelihoods based on 10 runs.

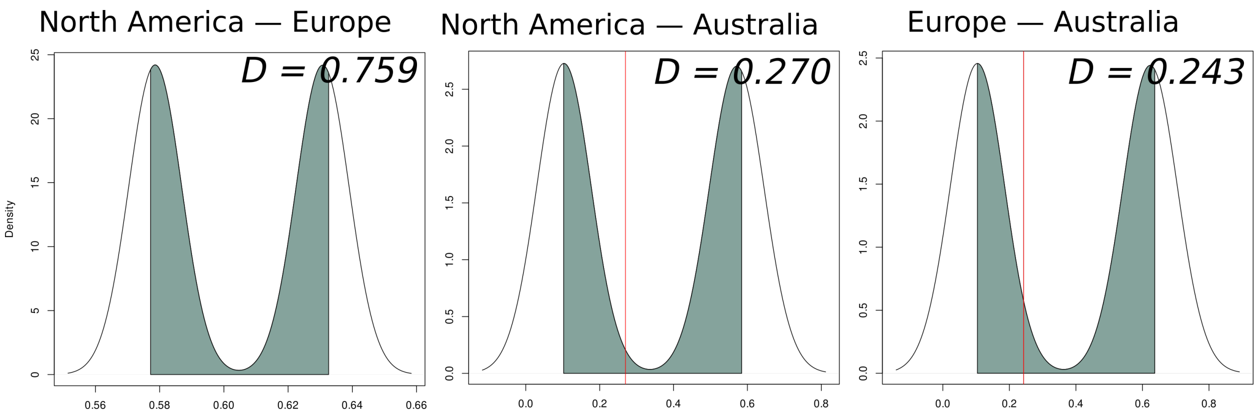

Figure S3. Distribution of schoeners *D* for between-range comparisons based on 200 random permutations. The shaded region is the middle 95%-ile of the null distribution. The observed *D* value is reported on the top-right of each panel. The shaded region is the middle 95%-ile of the null distribution. The red line indicates the observed *D* value and is absent if *D* fell outside the null distribution.

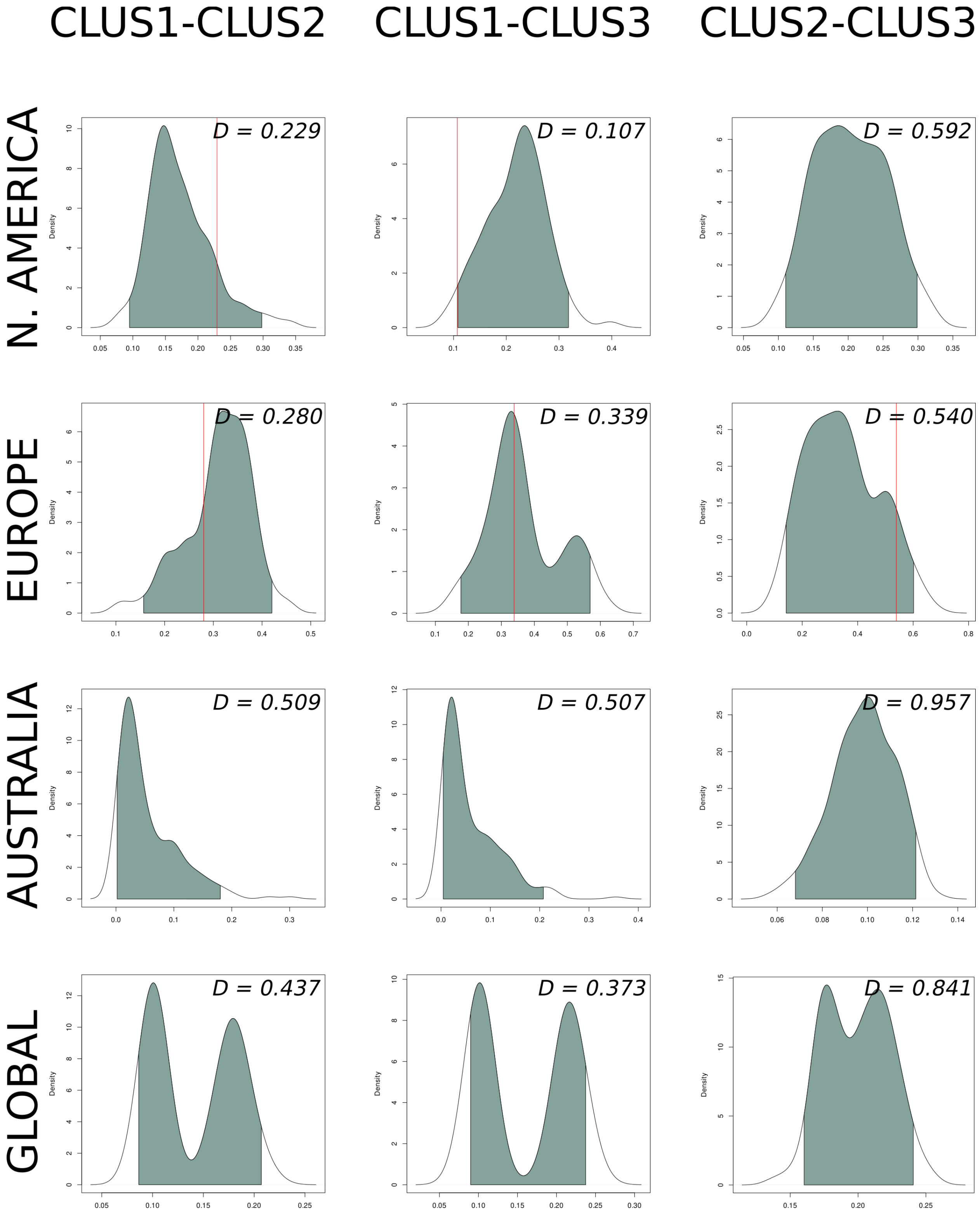

Figure S4. Distribution of Schoener's *D* for various cluster-range comparisons based on 200 random permutations. The observed *D* value is reported on the top-right of each panel. The shaded region is the middle 95%-ile of the null distribution. The red line indicates the observed *D* value and is absent if *D* fell outside the null distribution.

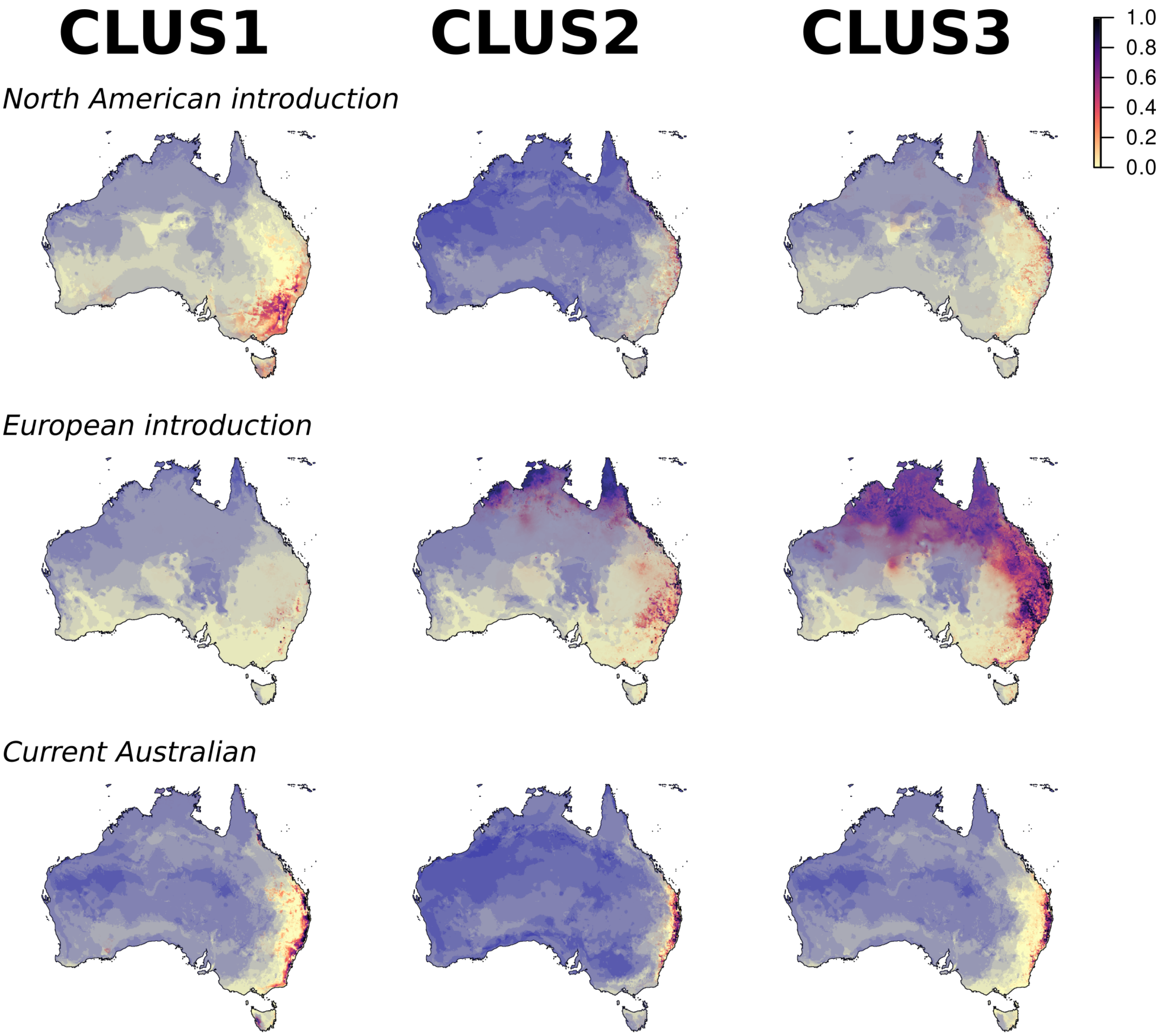

Figure S5. Mobility-oriented parity (MOP; blue) maps overlaid on the predicted distribution of ragweed under different invasion scenarios involving the introduction of a single cluster from different ranges to Australia. For each subplot, the opacity of the MOP map depends on the number of bioclimatic variables for which values in Australia fell outside the observed range. Higher opacity indicates a greater degree of model extrapolation.

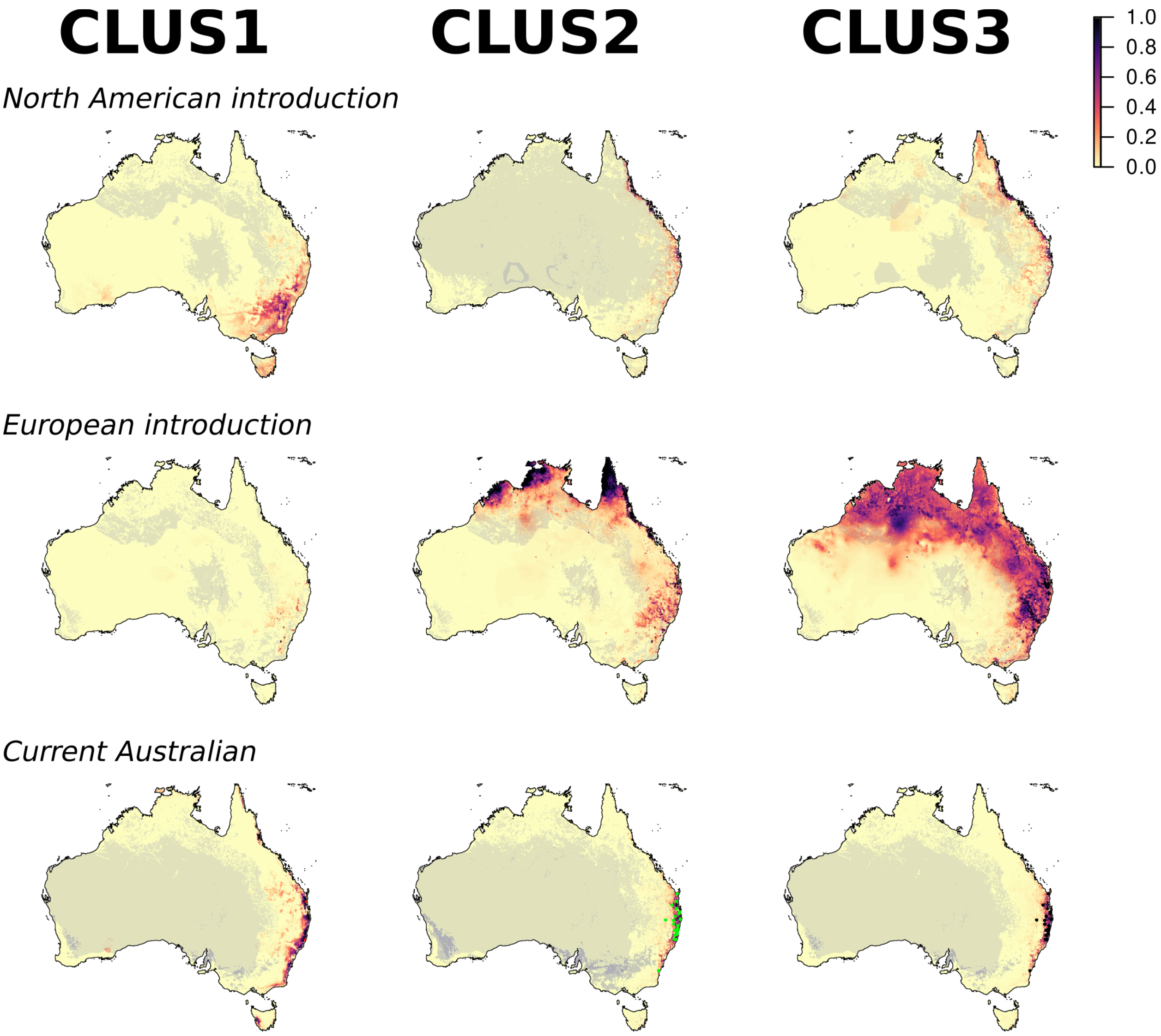

Figure S6. Mobility-oriented parity (MOP) maps overlaid on the predicted distribution of ragweed under different invasion scenarios involving the introduction of a single cluster from different ranges to Australia. For each subplot, the opacity of the MOP map depends on the number of land use/cover variables for which values in Australia fell outside the observed range. Higher opacity indicates a greater degree of model extrapolation.

We initially compared different ensemble niche models using the R\biomod2 package (Thuiller et al., 2021) in R-4.3.1**.** For each method, we fitted models to GBIF records from the given range and 10,000 randomly sampled pseudoabsences. We evaluated model performance by projecting models to Australia and estimating ROC values using Australian occurrence records and 10,000 randomly sampled Australian pseudoabsences (Table S1). Most methods performed similarly, with random forests performing the best. However, we opted to use maximum entropy models because they were also among the best-performing and is more straightforward to implement.

Table S1. Mean ROC for models trained on different ranges and validated in Australia.

**Method**	**Training Range**		
	North America	Europe	Australia
ANN (Artificial neural network)	0.906	0.852	-
CTA (Classification tree analysis)	0.939	0.874	0.9458000
FDA (Flexible discriminant analysis)	0.964	0.931	0.9848000
GAM (Generalised additive model)	0.966	0.926	0.8805556
GBM (Generalised boosting model)	0.968	0.933	0.9868000
GLM (Generalised linear model)	0.964	0.928	0.9456000
MARS (Multiple adaptive regression splines)	0.968	0.925	0.9460000
MAXNET (Maximum entropy)	0.967	0.925	0.9908000
RF (Random forest)	0.968	0.941	0.9932000
SRE (Surface range envelope)	0.765	0.725	0.6814000
XGBOOST (eXtreme Gradient Boosting Training)	0.964	0.935	0.9872000

Table S2. Description of environmental predictors used in modelling and mobility-oriented parity (MOP) analyses.

Predictor name	Predictor type	Description	Used to generate MOP maps?
BIO1	Bioclimatic	Annual mean temperature	N
BIO2	Bioclimatic	Mean diurnal range	N
BIO3	Bioclimatic	Isothermality	Y
BIO4	Bioclimatic	Temperature seasonality	N
BIO5	Bioclimatic	Maximum temperature of warmest month	N
BIO6	Bioclimatic	Minimum temperature of coldest month	N
BIO7	Bioclimatic	Temperature annual range	Y
BIO8	Bioclimatic	Mean temperature of wettest quarter	Y
BIO9	Bioclimatic	Mean temperature of driest quarter	Y
BIO10	Bioclimatic	Mean temperature of warmest quarter	N
BIO11	Bioclimatic	Mean temperature of coldest quarter	N
BIO12	Bioclimatic	Annual precipitation	Y
BIO13	Bioclimatic	Precipitation of wettest month	N
BIO14	Bioclimatic	Precipitation of driest month	Y
BIO15	Bioclimatic	Precipitation seasonality	Y
BIO16	Bioclimatic	Precipitation of wettest quarter	N
BIO17	Bioclimatic	Precipitation of driest quarter	Y
BIO18	Bioclimatic	Precipitation of warmest quarter	Y
BIO19	Bioclimatic	Precipitation of coldest quarter	N
CULTRF	Land use/cover	Rain-fed cultivated land (percent of grid cell)	Y
CULTIR	Land use/cover	Irrigated cultivated land (percent of grid cell)	Y
CULT	Land use/cover	Total cultivated land (percent of grid cell)	Y
FOR	Land use/cover	Forest land (percent of grid cell)	Y
GRS	Land use/cover	Grass/scrub/woodland (percent of grid cell)	Y
URB	Land use/cover	Built-up land (percent of grid cell)	Y
NVG	Land use/cover	Barren/very sparsely vegetated land (percent of grid cell)	Y
WATER	Land use/cover	Mapped water bodies (percent of grid cell)	Y

Table S3. Effect of increasing number of ancestral populations *L* on five-fold cross-validation error of ADMIXTURE performed on North American samples.

L	Cross-validation error
2	0.73578
3	0.73457
4	0.73464
5	0.73789
6	0.73850
7	0.74288
8	0.74977
9	0.75682
10	0.76538
11	0.77083
12	0.77749
13	0.78641

Table S4: Number of observations for each cluster-range combination

	Range of origin		
	North America	Europe	Australia
**Cluster identity**			
CLUS1	107	176	2
CLUS2	101	106	79
CLUS3	95	92	102

Table S5. Model AUC for models created using GBIF records with different training datasets and projected regions

	Observation Origin			
	Global	North America	Europe	Australia
**Range projected to:**				
Global	0.980	0.966	0.962	0.922
North America	0.954	0.968	0.931	0.917
Europe	0.893	0.868	0.932	0.866
Australia	0.975	0.987	0.985	0.997

Table S6. Mean values of bioclimatic and land use/cover variables for different clusters. Different letters indicate statistically significant differences in mean values based on a two-sample Welch's t-test at 95% confidence.

	CLUS1	CLUS2	CLUS3
**BIO1**	9.404^{A}	12.111^{B}	13.177^{C}
**BIO2**	10.616^{A}	10.569^{A}	10.591^{A}
**BIO3**	31.520^{A}	36.611^{B}	38.529^{C}
**BIO4**	875.074^{A}	739.113^{B}	679.413^{C}
**BIO5**	26.889^{A}	27.365^{B}	27.684^{B}
**BIO6**	-7.207^{A}	-3.156^{B}	-1.306^{C}
**BIO7**	34.096^{A}	30.521^{B}	28.990^{C}
**BIO8**	17.008^{A}	17.647^{AB}	17.982^{B}
**BIO9**	0.364^{A}	4.669^{B}	6.814^{C}
**BIO10**	19.892^{A}	20.928^{B}	21.317^{B}
**BIO11**	-1.686^{A}	2.707^{B}	4.569^{C}
**BIO12**	847.600^{A}	978.122^{B}	1043.253^{C}
**BIO13**	98.246^{A}	125.678^{B}	135.228^{C}
**BIO14**	46.028^{A}	41.759^{B}	42.941^{B}
**BIO15**	25.547^{A}	34.118^{B}	34.122^{B}
**BIO16**	272.614^{A}	345.870^{B}	369.865^{C}
**BIO17**	153.326^{A}	145.832^{A}	151.443^{A}
**BIO18**	258.049^{A}	323.874^{B}	338.059^{B}
**BIO19**	163.375^{A}	166.014^{AB}	175.429^{B}
**CULTRF**	36.043^{A}	25.122^{B}	20.626^{C}
**CULTIR**	5.479^{A}	2.628^{B}	2.268^{B}
**CULT**	41.522^{A}	27.750^{B}	22.894^{C}
**FOR**	29.546^{A}	45.496^{B}	51.054^{B}
**GRS**	17.230^{A}	17.961^{A}	21.135^{B}
**URB**	9.566^{A}	6.974^{A}	3.776^{B}
**NVG**	0.000^{A}	0.105^{B}	0.070^{AB}
**WATER**	2.136^{A}	1.714^{AB}	1.072^{B}

Table S7. Schoener's *D* estimate of niche overlap between cluster-ranges based on models created using 19 bioclimatic and 8 land use/cover variables (NA = North America, EU = Europe, AU = Australia).

	**CLUS1 NA**	**CLUS2 NA**	**CLUS3 NA**	**CLUS1 EU**	**CLUS2 EU**	**CLUS3 EU**	**CLUS1 AU**	**CLUS2 AU**	**CLUS3 AU**
**CLUS1 NA**	-	0.2292209	0.1071579	0.2123543	0.127162	0.1468827	0.3118139	0.1835172	0.1811673
**CLUS2 NA**		-	0.5919192	0.2005026	0.2403407	0.2247902	0.3010468	0.3335283	0.3299163
**CLUS3 NA**			-	0.2047425	0.3572842	0.3752501	0.217387	0.2316163	0.2325178
**CLUS1 EU**				-	0.2802535	0.3385482	0.1733654	0.1532931	0.1548962
**CLUS2 EU**					-	0.5395117	0.1778968	0.132061	0.1337359
**CLUS3 EU**						-	0.1823857	0.1691083	0.1720969
**CLUS1 AU**							-	0.5085297	0.5067729
**CLUS2 AU**								-	0.957293
**CLUS3 AU**									-

Table S8. Mean F_{ST} estimated between cluster-ranges using vcftools (NA = North America, EU = Europe, AU = Australia).

	**CLUS1 NA**	**CLUS2 NA**	**CLUS3 NA**	**CLUS1 EU**	**CLUS2 EU**	**CLUS3 EU**	**CLUS1 AU**	**CLUS2 AU**	**CLUS3 AU**
**CLUS1 NA**	-	0.0010508	0.0013869	0.0024734	0.0015793	0.0021068	-0.23846	0.024016	0.02861
**CLUS2 NA**		-	-0.0001007	0.0015622	0.0014585	0.0024411	-0.24244	0.019992	0.023942
**CLUS3 NA**			-	0.0016346	0.0015246	0.0018794	-0.23718	0.023382	0.02846
**CLUS1 EU**				-	0.00064324	0.0019748	-0.24252	0.027101	0.032597
**CLUS2 EU**					-	0.00049948	-0.24338	0.027352	0.032281
**CLUS3 EU**						-	-0.24302	0.026728	0.031712
**CLUS1 AU**							-	-0.10984	-0.10255
**CLUS2 warmerAU**								-	-0.00026923
**CLUS3 AU**									-

References

Thuiller W, Georges D, Gueguen M, Engler R, Breiner F, Lafourcade B, Patin R (2023). *biomod2: Ensemble Platform for Species Distribution Modeling*. R package version 4.2-4, <https://CRAN.R-project.org/package=biomod2>.
